# High‐Performing Flexible Mg_3_Bi_2_ Thin‐Film Thermoelectrics

**DOI:** 10.1002/advs.202409788

**Published:** 2024-10-01

**Authors:** Boxuan Hu, Xiao‐Lei Shi, Tianyi Cao, Siqi Liu, Min Zhang, Wanyu Lyu, Liangcao Yin, Tuquabo Tesfamichael, Qingfeng Liu, Zhi‐Gang Chen

**Affiliations:** ^1^ School of Chemistry and Physics ARC Research Hub in Zero‐emission Power Generation for Carbon Neutrality and Centre for Materials Science Queensland University of Technology Brisbane Queensland 4000 Australia; ^2^ State Key Laboratory of Materials‐Oriented Chemical Engineering College of Chemical Engineering Nanjing Tech University Nanjing 211816 China; ^3^ School of Mechanical Medical and Process Engineering Queensland University of Technology Brisbane Queensland 4001 Australia

**Keywords:** device, flexibility, Mg_3_Bi_2_, thermoelectric, thin film

## Abstract

With the advances in bulk Mg_3_Bi_2_, there is increasing interest in pursuing whether Mg_3_Bi_2_ can be fabricated into flexible thin films for wearable electronics to expand the practical applications. However, the development of fabrication processes for flexible Mg_3_Bi_2_ thin films and the effective enhancement of their thermoelectric performance remain underexplored. Here, magnetron sputtering and ex‐situ annealing techniques is used to fabricate flexible Mg_3_Bi_2_ thermoelectric thin films with a power factor of up to 1.59 µW cm^−1^ K^−2^ at 60 °C, ranking as the top value among all reported n‐type Mg_3_Bi_2_ thin films. Extensive characterizations show that ex‐situ annealing, and optimized sputtering processes allow precise control over film thickness. These techniques ensure high adhesion of the films to various substrates, resulting in excellent flexibility, with <10% performance degradation after 500 bending cycles with a radius of 5 mm. Furthermore, for the first time, flexible thermoelectric devices are fabricated with both p‐type and n‐type Mg_3_Bi_2_ legs, which achieve an output power of 0.17 nW and a power density of 1.67 µW cm^−2^ at a very low temperature difference of 2.5 °C, highlighting the practical application potential of the device.

## Graphical abstract

A method to tune the p‐ and n‐type Mg_3_Bi_2_ thin films using magnetron sputtering followed by ex‐situ annealing. The reactions occurring during the ex‐situ annealing are analyzed, and the thin film achieve a power factor of 1.59 µW cm^−1^ K^−2^ and a *ZT* value of 0.19 at 60 °C.

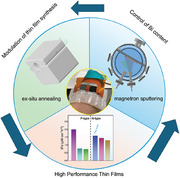

## 1 Introduction

Thermoelectric technology is positioned as a promising green energy solution that directly converts waste heat into electrical energy. Flexible thermoelectric devices, in particular, are emerging as a promising sustainable power source for wearable electronics, including smart glasses, watches, wireless headphones, e‐skins, and smart rings.^[^ ^1^, ^2^, ^3^, ^4^, ^5^, ^6^, ^7^, ^8^ ^]^ The efficiency of a thermoelectric device is highly correlated to the performance of thermoelectric materials, described by the dimensionless figure‐of‐merit *ZT*, determined by *ZT* = *S^2^σT*/*κ*, where *S*, *σ*, *T*, and *κ* are the Seebeck coefficient, the electrical conductivity, the absolute temperature, and the thermal conductivity, respectively. *S^2^σ* stands for the power factor,^[^ ^9^, ^10^, ^11^ ^]^ and *κ* is comprised of the electronic thermal conductivity *κ*_e_ and the lattice thermal conductivity *κ*_l_. To optimize the *ZT*, the main strategy is to enhance the *S* and the *σ* while reducing the *κ*. The *S*, *σ*, and *κ*_e_ are highly correlated with the carrier concentration *n*. Typical strategies for tuning the *n* include rational doping and alloying.^[^ ^12^ ^]^ Doping methods can effectively induce the point defects to tune the *n* and *S*.^[^ ^12^ ^]^ Alloying with the second phase can stimulate the energy filtering effect to improve the *S* while maintaining high *σ*.^[^ ^13^ ^]^ The main strategy to reduce the *κ*_l_ is typically inducing crystal or lattice defects as additional scattering centers to enhance phonon scattering. However, the induced defects can also scatter charge carriers, leading to a reduction of the carrier mobility *µ* and in turn, the *σ*.^[^ ^2^ ^]^ Therefore, optimizing the performance of thermoelectric materials still presents a challenge due to the trade‐off of each parameter.^[^ ^14^ ^]^

Bi_2_Te_3_ and Bi_0.5_Sb_1.5_Te_3_, as the most used near‐room‐temperature thermoelectric materials,^[^ ^15^ ^]^ are widely applied in flexible thermoelectric device designs due to their high *ZT* values.^[^ ^2^, ^15^, ^16^, ^17^ ^]^ However, its high cost, particularly the expensive price of Te,^[^ ^11^ ^]^ limits its potential for large‐scale commercial use.^[^ ^17^, ^18^, ^19^ ^]^ Some newly developed near‐room‐temperature thermoelectric materials, such as silver chalcogenides (Ag_2_Q, where Q = S, Se, Te),^[^ ^20^, ^21^, ^22^ ^]^ offer thermoelectric performance similar to Bi_2_Te_3_ and notable plasticity, but Ag is relatively expensive. In contrast, the recently popular thermoelectric material, Mg_3_Bi_2_, can effectively reduce the cost of thermoelectric materials by minimizing the need for costly Te and Ag.^[^ ^23^, ^24^ ^]^ Mg‐based alloys offer abundant availability, low cost, and low toxicity, making them more suitable for large‐scale production of thermoelectric devices.^[^ ^25^, ^26^, ^27^, ^28^, ^29^, ^30^, ^31^, ^32^, ^33^, ^34^ ^]^ Particularly, the recently reported Mg_3_Bi_2_ crystals exhibit high plasticity,^[^ ^19^, ^24^, ^35^ ^]^ making them promising for flexible thermoelectric materials and devices. As a result, there is significant interest in whether the excellent thermoelectric performance and plasticity of Mg_3_Bi_2_ can be realized in thin films.

However, while research on bulk Mg_3_Bi_2_ has matured, studies on its thin‐film counterparts remain limited.^[^ ^36^ ^]^ Achieving high thermoelectric performance in Mg_3_Bi_2_ thin films remains challenging compared to the high *ZT* of 0.9 achieved with bulk Mg_3_Bi_2_ at room temperature.^[^ ^18^ ^]^ Current research on Mg_3_Bi_2_ thin films has not realized high *S* ^2^ *σ*. Reports indicate that p‐type Mg_3_Bi_2_ thin films epitaxially grown on *c*‐plane sapphire substrates achieved an *S* ^2^ *σ* of 2 µW cm⁻^1^ K⁻^2^ near room temperature.^[^ ^37^ ^]^ Additionally, polycrystalline p‐type Mg_3_Bi_2_ thin films with in situ annealing showed a maximum *S* ^2^ *σ* of 0.89 µW cm⁻^1^ K⁻^2^.^[^ ^38^ ^]^ However, compared to bulk Mg_3_Bi_2_ materials, the performance of Mg_3_Bi_2_ thin films is relatively lower, and the complex fabrication processes limit their practical application. At the same time, Mg‐based thermoelectric thin films are sensitive to various characterization methods, and most reports lack clear microscopic characterization to explain the relationship between the performance and structure of Mg_3_Bi_2_ thin films. Furthermore, p‐type Mg_3_Bi_2_ thin films have been reported, but there are still few reports on the realization of high‐performance n‐type Mg_3_Bi_2_ thin films. Therefore, there is still considerable room to enhance the practical application potential of Mg_3_Bi_2_ thin films, particularly n‐type films, by improving their thermoelectric performance and stability or further simplifying the manufacturing processes.

**Figure 1:**
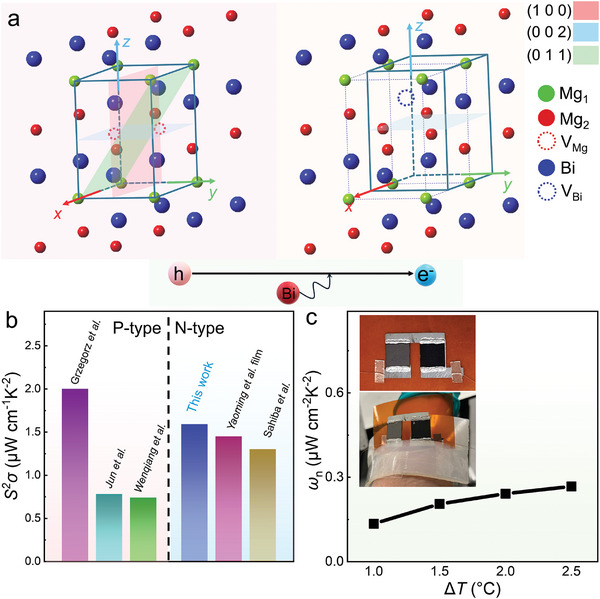
Introduction of Mg_3_Bi_2_ thin films prepared by magnetron sputtering and *ex‐site* annealing. a) Unit cells of Mg_3_Bi_2_ from p‐type to n‐type with highlighted (1 0 0), (0 0 2), and (0 1 1) planes. b) Comparison of power factor (*S* ^2^ *σ*) of both p‐type and n‐type Mg_3_Bi_2_ thin films over the past five years.^[^ ^37^, ^38^, ^39^, ^40^, ^41^ ^]^ c) Normalized power density (*ω*_n_) of the wearable devices at different temperature differences (Δ*T*) prepared in this work with the insets displaying photographs of wearing the device for power generation.

Compared to previously reported Mg_3_Bi_2_ thin films prepared by magnetron sputtering,^[^ ^37^, ^38^, ^39^, ^40^, ^41^ ^]^ our goal is to design a simpler synthesis method to achieve either p‐type or n‐type thin‐film materials by simply controlling the Bi composition. To achieve this, we propose a method that combines dual‐target co‐sputtering and ex‐situ annealing to deposit Mg_3_Bi_2_ thin films on polycrystalline Al_2_O_3_ substrates and then flexible polyimide (PI) substrates. By adjusting the deposition power of the Bi target, we control the Bi content in the films to achieve n‐type or p‐type semiconductor characteristics. The ex‐situ annealing process promotes the bonding of Mg and Bi, forming Mg_3_Bi_2_ thin films. The detailed fabrication process is illustrated in Figure S1 (Supporting Information). **Figure** **1**a shows the crystal structures of p‐type and n‐type ex‐situ annealed films. When Mg is abundant, the material exhibits Mg vacancies (V_Mg_), leading to hole conduction. With increasing the Bi content, Bi vacancy (V_Bi_) appears, and the material exhibits electron conduction behavior. Additionally, after ex‐situ annealing, p‐type films show three strong peaks characteristic of bulk Mg_3_Bi_2_: (100), (002), and (011), while n‐type films display a strong (002) preferred orientation (see XRD results analysis later). We also found that different annealing temperatures affect the bonding of Mg and Bi, with reactions occurring only when the temperature is close to the melting point of Bi (271.5 °C). A 15‐h annealing process at 265 °C further improved the crystallinity of the films. Using this strategy, we obtained stable n‐type Mg_3_Bi_2_ thin films with an *S* ^2^ *σ* value of 1.59 µW cm^−1^ K^−2^, surpassing most previously reported values,^[^ ^37^, ^38^, ^39^, ^40^, ^41^ ^]^ as shown in Figure 1b. Specifically, our performance is highest in the n‐type films, with *S* ^2^ *σ* value that is only slightly lower than the reported highest values for p‐type films. Furthermore, to validate the applicability of our designed films, we designed a thermoelectric device with a p‐n junction consisting of a pair of Mg_3_Bi_2_‐based thin films using a modular design. The normalized power density (*ω*_n_) of this device reached 0.27 µW cm^−2^ K^−2^ at a temperature difference (Δ*T*) of 2.5 °C, as shown in Figure 1c. The device was fabricated by depositing Ag electrodes and wires on PI using electron beam evaporation, and then fixing the ends of the films to the electrodes with thermosetting silver paste, as illustrated in the inset of Figure 1c. Therefore, the flexible device shows significant potential for practical wearable applications.

## 2 Results and Discussion

**Figure 2:**
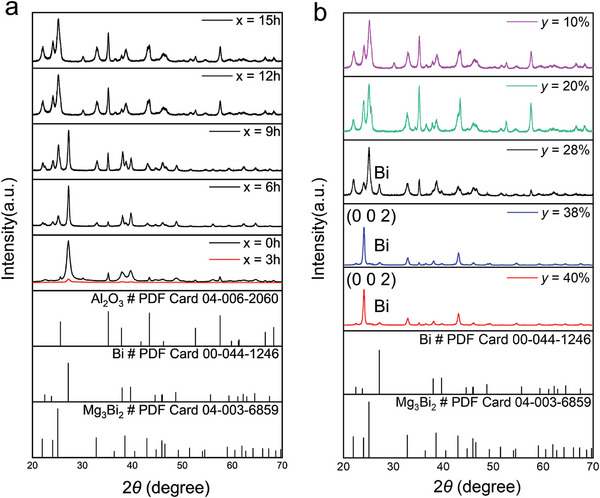
Grazing incidence X‐ray diffraction (GIXRD) patterns of Mg_3_Bi_2_ thin films. a) GIXRD patterns of Mg_3_Bi_2_ thin films with different annealing time (*x*). b) GIXRD patterns of Mg_3_Bi_2_ thin films with different Bi content (*y*). The 2*θ* ranges from 20 to 70°.

In this work, we utilized magnetron sputtering to fabricate Mg_3_Bi_2_ thin films. Magnetron sputtering stands out for its ability to deposit uniform, high‐performance, and well‐adhered inorganic films on various substrates. Consequently, it has been widely adopted for manufacturing thin‐film thermoelectric materials. This technique involves sputtering target material atoms onto the substrate surface through ion bombardment in a room‐temperature argon environment. By adjusting sputtering parameters such as pressure and deposition power, the thermoelectric performance, thickness, and flexibility of the films can be optimized, allowing for the production of inorganic thermoelectric films with a range of characteristics and structures.^[^ ^42^ ^]^ Therefore, magnetron sputtering is considered one of the most effective methods for fabricating large‐area, high‐performance thermoelectric thin films.^[^ ^42^, ^43^ ^]^ To further investigate the reaction process of Mg and Bi during the ex‐situ annealing, we varied the annealing time (*x*) to 0, 3, 6, 9, 12, and 15 h, and measured the grazing incidence X‐ray diffraction (GIXRD) patterns of these samples. **Figure** **2**a presents the GIXRD patterns of Mg_3_Bi_2_ thin films within the 2*θ* range of 20 to 70°. Due to the use of a polycrystalline Al_2_O_3_ substrate, all samples show Al_2_O_3_ peaks. When *x* = 0, the unannealed film exhibits a strong Bi peak but no Mg peak, indicating the presence of amorphous Mg and crystalline Bi before annealing. At *x* = 3, the intensity of the Bi peak starts to decrease, while the Mg_3_Bi_2_ peak is still absent. This is because the annealing temperature of 265 °C is close to the melting point of Bi, which weakens the crystallinity of Bi. As *x* increases to 6 h, the Mg_3_Bi_2_ peak appears in the film, indicating that the binding of Mg and Bi began between 3 and 6 h of annealing. However, Bi remains the predominant crystalline component. At *x* = 9 h, the content of Mg_3_Bi_2_ crystals further increases, replacing Bi as the main crystalline component. At *x* = 12 h, no Bi crystalline peaks are observed in the film. Similarly, at *x* = 15 h, the XRD results resemble those of the film annealed for 12 h, suggesting that all Bi has either reacted with Mg or remains in an amorphous state, indicating that the reaction is nearing completion. A more detailed description of this process is provided in Figure S2 (Supporting Information).

To investigate whether the crystal composition of the material changes with varying Bi content, we prepared a series of thin films with different Bi composition ratios, denoted as *y*. The overall composition of the film is Mg_3_Bi_(3_ *_y_*_/1−_ *_y_*_)_. GIXRD patterns were explored, as shown in Figure 2b. When the *y* is 10% or 20%, the patterns only display Mg_3_Bi_2_ crystalline features, indicating that additional Bi does not participate in the crystallization process. As *y* increases to 28%, Bi crystalline peaks begin to appear, suggesting that Bi at this level does not fully react with Mg. Based on previous analysis, the Bi crystals may form from unreacted molten Bi that recrystallizes during the annealing and cooling process. When the *y* is further increased to 38%, Mg_3_Bi_2_ shows strong (0 0 2) orientation along with some weak Bi peaks. Thus, when the *y* is 10% or 20%, Bi fully reacts with Mg. Conversely, at *y* = 38% and 40%, the excess Bi content indicates that some Bi does not participate in the reaction. Moreover, excess Bi may directly affect the crystallinity of the film, inducing (0 0 2) preferred orientation.

**Figure 3:**
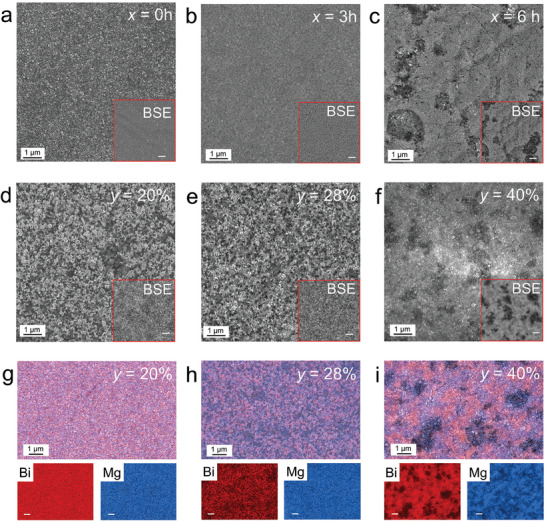
Morphological and compositional characteristics of Mg_3_Bi_2_ thin films. a–c) Scanning electron microscopy (SEM) images of Mg_3_Bi_2_ thin films annealing under *x* = 0, 3, and 6 h with Bi content *y* = 40%. The insets are the corresponding backscattered electron (BSE) images. d‐f) SEM images of Mg_3_Bi_2_ thin films with Bi content *y* = 20%, 28%, and 40% under annealing time *x* = 15 h. The insets are the BSE images. g–i) Energy‐dispersive X‐ray spectroscopy (EDS) mapping images of Mg_3_Bi_2_ thin films with Bi content *y* = 20%, 28%, and 40%, under *x* = 15 h. Red represents Bi, blue represents Mg, and white scale bars all represent 1 µm.

To investigate the structural changes during ex‐situ annealing and the impact of different Bi contents on the structure and composition of Mg_3_Bi_2_ thin films, we employed scanning electron microscopy (SEM) equipped with the energy dispersive X‐ray spectrometry (EDS) to study the surface morphology and elemental distribution of the films at various annealing times. **Figure** **3**a–c display the SEM images of Mg_3_Bi_2_ films with *y* = 40% annealed for 0, 3, and 6 h, respectively. As shown in Figure 3a, the unannealed film surface is dense. At *x* = 3 h, Bi starts to transit to an amorphous state, but the film surface, as seen in Figure 3b, remains relatively dense. Figure 3c reveals that after *x* = 6 h, the film surface exhibits wrinkles and some voids. This is attributed to molten Bi, which, influenced by airflow within the tube furnace, moves and carries some Mg inside. Insets in Figure 3a–c are backscattered electron (BSE) images, indicating that the elemental distribution becomes more heterogeneous with increasing the annealing time.

Figure 3d–f show SEM images of films annealed for 15 h with *y* values of 20%, 28%, and 40%. Compared to the unannealed film, the film with *y* = 20% exhibits a lower density, as shown in Figure 3d. With increasing *y*, the 28% Bi film surface becomes rougher and further reduces density. At *y* = 40%, the film surface shows several voids, significantly impacting density. Insets in Figure 3d–f are BSE images, revealing a more pronounced uneven distribution of elements with decreasing density. This unevenness is likely due to the higher Bi content, which causes more Mg to move with molten Bi under the influence of airflow in the tube furnace. To further analyze the elemental distribution, Figure 3g–i present EDS images of films with *y* values of 20%, 28%, and 40% after 15 h of annealing. When *y* = 20%, the distribution of Mg and Bi is uniform, with no noticeable elemental segregation. At *y* = 28%, Mg distribution remains uniform, but there are slight Bi clusters, though the overall distribution is still relatively even. At *y* = 40%, both Mg and Bi show noticeable aggregation, resulting in an uneven distribution. This is consistent with the SEM images and the analysis results. Additional SEM and corresponding EDS data for samples with varying Bi content and annealing times are provided in Figures S3–S18 (Supporting Information).

**Figure 4:**
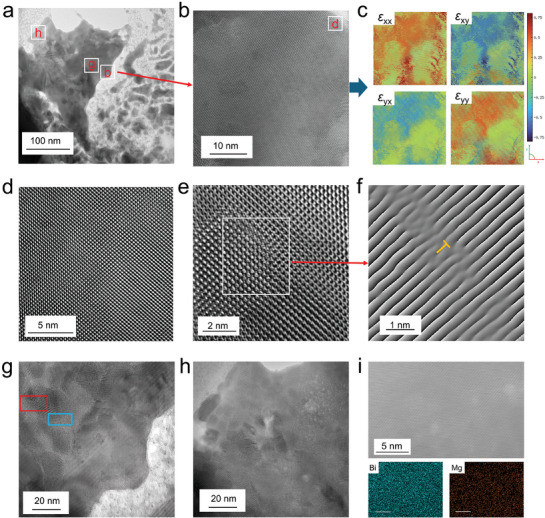
Micro/nanostructural features of Mg_3_Bi_2_ thin films with Bi content *y* = 40%. a) Low magnification transmission electron microscopy (TEM) image of the samples prepared using focused ion beam (FIB) technology. b) High‐resolution TEM (HRTEM) image taken from a normal region. c) Corresponding strain maps taken in different directions. d) HRTEM image taken from the thinner region from b. e) HRTEM image showing defects near area d. f) Filtered image extracted from e, indicating the possible presence of edge dislocations. g) HRTEM image of the highly distorted region from a, showing potential coherent (blue) and incoherent (red) boundaries within the selected area. h) HRTEM images of the Mg_3_Bi_2_ and amorphous Mg interface from a. i) High‐angle annular dark field (HAADF) image of the Mg_3_Bi_2_, along with the corresponding EDS mapping.

To analyze the micro/nanostructures and detailed compositions of Mg_3_Bi_2_ thin films, we utilized focused ion beam (FIB) technology to prepare transmission electron microscopy (TEM) samples, which were then characterized using aberration‐corrected scanning transmission electron microscopy (Cs‐STEM). **Figure** **4**a displays a low‐magnification TEM image of a Mg_3_Bi_2_ thin‐film sample with Bi content *y* = 40%. The image reveals aggregates of Mg_3_Bi_2_ crystals encased in amorphous Mg. Figure 4b shows a high‐resolution TEM (HRTEM) image taken from a normal region of Figure 4a, with the corresponding fast Fourier transform (FFT) pattern shown in Figure S19 (Supporting Information). This confirms that the observation direction of the FIB sample is along the [1 0 0] direction. The observation plane of the sample is perpendicular to the film, allowing the (0 0 2) spots to be visible in this image, which is consistent with the XRD data. While the lattice arrangement in the selected area is generally ordered, the image reveals multiple lattice distortions. Figure 4c presents the corresponding strain maps taken from Figure 4b, indicating significant strain along the *x*‐direction, which may contribute to lattice distortions. Figure 4d, taken from a thinner region in Figure 4b, shows lattice arrangement in defect‐free regions of the Mg_3_Bi_2_ crystals. Figure 4e shows another region near Figure 4d with strong contrast differences, indicating notable lattice distortions. Figure 4f provides a filtered image corresponding to Figure 4e, revealing potential edge dislocations within the film. Figure 4g is an enlarged view of the highly distorted area in Figure 4a, displaying coherent (blue) and incoherent (red) boundaries within the selected range, indicating more pronounced two‐dimensional defects. Figure 4h is an enlarged view of the Mg_3_Bi_2_ and amorphous Mg interface in Figure 4a, showing the presence of amorphous Mg as a secondary phase, which may negatively impact the optical and electrical properties of the material. Figure 4i displays a Cs‐STEM HAADF image of the Mg_3_Bi_2_ crystal regions and the corresponding EDS map. Overall, the elemental distribution in the Mg_3_Bi_2_ crystal regions is uniform on a microscale. Combined with the information on amorphous Mg regions shown in Figures S20–S21 (Supporting Information) and the overall low‐magnification EDS data, we hypothesize that the sample contains a substantial amount of amorphous pure Mg encasing Mg_3_Bi_2_ crystals. Detailed lattice calibration information can be found in Figures S22–S24 (Supporting Information).

**Figure 5:**
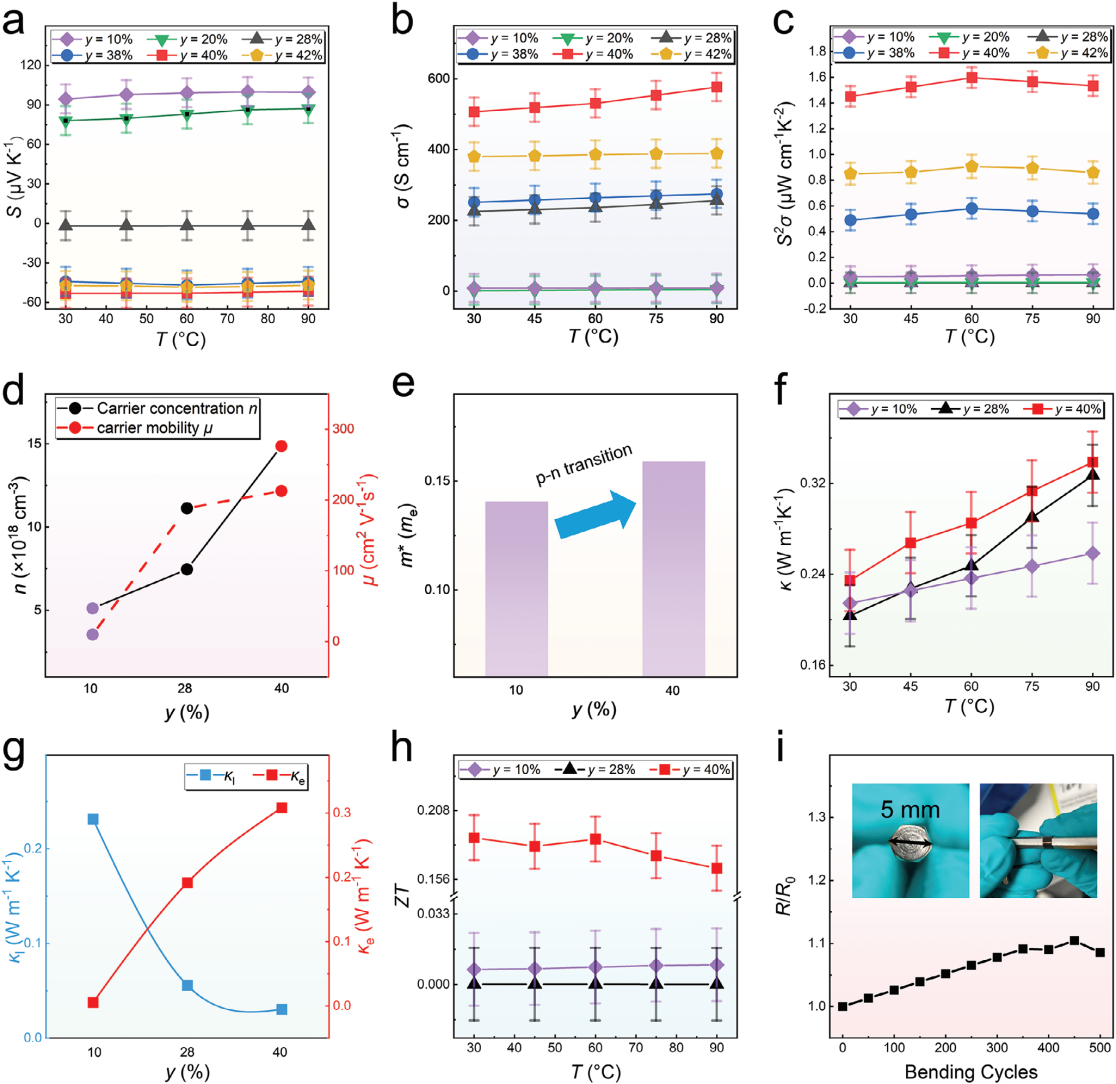
Thermoelectric performance and flexibility of Mg_3_Bi_2_ thin films with different Bi contents *y* (*y* = 10%, 20%, 28%, 38%, 40%, 42%). a) Seebeck coefficient (*S*), b) electrical conductivity (*σ*), and c) *S* ^2^ *σ* of Mg_3_Bi_2_ thin films. d) Room‐temperature carrier concentration (*n*) and mobility (*µ*). e) Effective mass (m*) calculated using the single parabolic band (SPB) model. f) Temperature‐dependent thermal conductivity (*κ*). g) Lattice thermal conductivity (*κ*_l_) and electrical thermal conductivity (*κ*_e_), and h) Estimated temperature‐dependent figure‐of‐merit *ZT*. i) Resistance change (*R*/*R*_0_) of Mg_3_Bi_2_ thin films with Bi content *y* = 40% on a PI substrate under different bending cycles with a bending radius of 5 mm.

To investigate the specific impact of varying Bi content (*y*) on the thermoelectric performance of Mg_3_Bi_2_ films, we measured the thermoelectric properties of Mg_3_Bi_2_ films with Bi contents of *y* = 10%, 20%, 28%, 38%, 40%, and 42%. The film thickness, measured using a profilometer, is ≈400 nm, with detailed information provided in Figures S25–S27 (Supporting Information). **Figure** **5**a–c shows the temperature‐dependent *S*, *σ*, and *S* ^2^ *σ* for these films. As can be seen in Figure 5a, the samples with *y* = 10% and 20% exhibit positive *S*, indicating a p‐type semiconducting behavior. Conversely, samples with *y* = 38%, 40%, and 42% show negative *S*, characteristic of an n‐type semiconducting behavior. At ≈28% Bi content, the *S* approaches zero, suggesting this Bi concentration is near the p‐n transition point. For p‐type materials, the Mg_3_Bi_2_ film with *y* = 10% has a high *S* of 100 µV K^−1^. As the Bi content increases to 20% and 28%, the *S* starts to decrease. For n‐type materials, the film with *y* = 40% exhibits the highest *S* of −53 µV K^−1^. The higher *S* in p‐type materials is attributed to changes in the binding energy between Mg and Bi as Bi concentration varies. Increased Bi content facilitates the formation of Mg_3_Bi_2_ with V_Bi_. As the *y* value increases for n‐type Mg_3_Bi_2_ films, the *S* gradually decreases. When the *y* is ≈28%, the *S* for n‐type and p‐type materials are nearly equal, resulting in an overall *S* close to zero. Further increasing *y* leads to dominant n‐type behavior, but excessive Bi can cause self‐crystallization of Bi, reducing the amount of n‐type material and consequently lowering the *S*.

As shown in Figure 5b, the *σ* increases from 5 to 506 S cm^−1^ at 303 K for *y* = 10% to 40%, attributed to the increased *n*, as discussed later. When the *y* exceeds 40%, the *σ* begins to decrease. As shown in Figure 2b, Bi peaks are already visible in the XRD pattern when the material transitions from p‐type to n‐type. Additionally, when *y* is greater than 40%, the self‐cohesion of Mg reduces the availability of Mg to fully participate in forming the Mg_3_Bi_2_ compound. As a result, there are more complex scattering interfaces between Mg─Mg, Mg─Bi, and Bi─Bi compared to other components, leading to a noticeable decrease in *σ*. At lower Bi contents (*y*), a significant amount of amorphous Mg is present, reducing the *σ*. As the Bi content increases, the amount of amorphous Mg decreases, leading to an increase in *σ*. When the material becomes n‐type, excess Bi starts to crystallize, forming Bi crystals that further reduce *σ*. At *y* = 40%, the proportion of amorphous material is minimized, resulting in the highest *σ*. Consequently, the film with *y* = 40% exhibits the highest *S* ^2^ *σ*. At 60 °C, S reaches −53 µV K^−1^, *σ* reaches 530 S cm^−1^, and *S* ^2^ *σ* reaches 1.59 µW cm^−1^ K^−2^. The measurement method using the ZEM‐3 is shown in Figure S28 (Supporting Information).

To investigate the variations in *σ* and *S*, we measured the *µ* and *n* at room temperature for the materials with the highest *S* ^2^ *σ* in the p‐type (*y* = 10%), n‐type (*y* = 40%), and the material at the p‐n transition point (*y* = 28%), as shown in Figure 5d. The n‐type thin film exhibits the highest *µ* and *n*, with a *µ* reaching 212.82 cm^2^ V^−1^ s^−1^ and *n* at 1.49 × 10^19^ cm⁻^3^. As the *y* decreases, both *µ* and *n* decline. Moreover, the *n* for our materials is lower than the reported optimal *n* of 3.6 × 10^19^ cm⁻^3^,^[^ ^18^ ^]^ which may result in the relatively low *σ*. This finding aligns with our earlier hypothesis that the material contains a substantial number of defects and secondary phases, with higher and lower *y* increasing the formation of secondary phases, thus further reducing the *σ* and *n*. To explain the variations in the *n* and *µ* of the thin films, the effective mass (*m* ^*^) data at room temperature for these thin films were calculated using the single parabolic band (SPB) model, as shown in Figure 5e. For p‐type and n‐type Mg_3_Bi_2_ thin films, the change in the *m* ^*^ is not particularly significant, with the n‐type material exhibiting slightly higher *m* ^*^ than the p‐type material. This is because the p‐type material has a lower *n* than the n‐type material, the absolute value of *S* for the p‐type material is higher, resulting in similar *m* ^*^ values for both.

The thermal diffusivity (*D*) of the thin films was determined using the laser flash method (PIT) with alternating current technique, and then we can achieve the *κ* data based on measured *D* values. The testing methods and principles for measuring *D* of the thin film are described in Figure S29 (Supporting Information). Through this method, we obtained relatively reliable *κ* data for the Mg_3_Bi_2_ thin films, as shown in Figures S30–S32 (Supporting Information). The *κ* of the thin films is relatively low with values ranging from 0.2 to 0.23 W m^−1^ K^−1^, as shown in Figure 5f. Figure 5g compares the *κ*_e_ and *κ*_l_ of the Mg_3_Bi_2_ thin films. *κ*_e_ can be calculated using the formula *κ*_e_ = *LσT*,^[^ ^44^ ^]^ where *L* is the Lorenz constant for Mg_3_Bi_2_. We determined the *L* for the Mg_3_Bi_2_ thin films using the SPB model (see Figure S33, Supporting Information), and *κ*_l_ can be calculated by subtracting *κ*_e_ from *κ*. Due to the various dimensional defects present in our Mg_3_Bi_2_ thin film samples (as analyzed in the TEM images), the *κ*_l_ of the Mg_3_Bi_2_ thin film is < 0.25 W m⁻^1^ K⁻^1^. Meanwhile, the *κ*_e_ of these samples are also low due to the low *σ*. Thus, the thin film with *y* = 40% can still achieve a maximum *ZT* of 0.19 at 60 °C, as shown in Figure 5h.

To demonstrate the flexibility potential of the thin films prepared by magnetron sputtering and ex‐situ annealing, we deposited a Mg_3_Bi_2_ thin film with *y* = 40% on PI and conducted 500 bending tests with a bending radius *r* of 5 mm. The results are shown in Figure 5i. The inset in Figure 5i shows the bending test process and method. The results indicate that the resistance change (*R*/*R*_0_) at *r* = 5 mm remained within 10%, the SEM comparative information before and after bending is shown in Figure S34 (Supporting Information), demonstrating that the Mg_3_Bi_2_ thin films prepared by this process have excellent flexibility potential and good adhesion to the flexible PI substrate.

**Figure 6:**
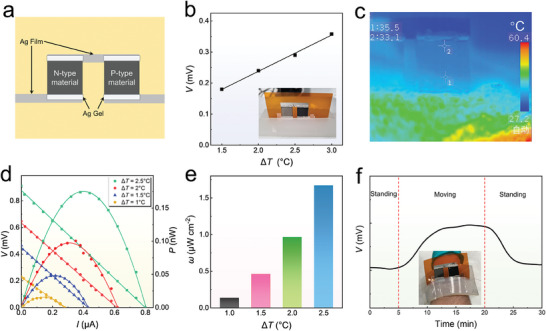
Design and performance of Mg_3_Bi_2_ thin‐film‐based flexible thermoelectric device. a) Schematic diagram of the modular device design. b) Voltage (*V*) of the device at temperature differences (Δ*T*s) = 1.5, 2, 2.5, and 3 °C. The inset shows a photograph of the device during testing. c) Infrared Δ*T* image of the device during testing. d) Relationship between *V*, current (*I*), and power (*P*) of the device at Δ*T*s of 1, 1.5, 2, and 2.5 °C. e) Power density (*ω*) of the device at Δ*T*s of 1, 1.5, 2, and 2.5 °C. f) Variation in *V* of the device when the wearer is in standing and moving states. The inset shows a photograph of the device while being worn.

To demonstrate the potential practical application of the materials prepared by this method, we designed a flexible thermoelectric device with a pair of legs, as illustrated in **Figure** **6**a. The device was fabricated using a modular design consisting of electron beam‐deposited Ag electrodes and wire modules, Mg_3_Bi_2_ thin film modules, Ag gel adhesion modules, and a silicone gel base module. Figure 6b shows that the device can generate a voltage (*V*) of 0.36 mV at a Δ*T* of 3 K, with the inset showing a photograph of the device. Figure 6c displays the infrared temperature difference image during testing, confirming the accuracy of the measurements. Figure 6d,e illustrates the current (*I*), *V*, output power (*P*), and power density (*ω*) by the device under different Δ*T*s. The device achieves a maximum *V* of 0.9 mV, *P* of 0.17 nW, and *ω* of 1.67 µW cm^−2^ at a Δ*T* of 2.5 K. Additionally, after multiple bending cycles (*r* = 10 cm), the *R*/*R*_0_ of the device remains below 10%, as shown in Figure S35 (Supporting Information). To evaluate the stability of the device, we remeasured it after one month and found that its *R*/*R*_0_ had decreased by 15.3%, as shown in Figure S36 (Supporting Information). This degradation is likely due to the inevitable decomposition of Mg‐based materials over time when exposed to air.^[^ ^18^ ^]^ Therefore, the device adopts a modular design, allowing for replacement after material decomposition. To verify the practicality of the modular thermoelectric device, we attached it to the surface of a human arm and conducted power generation tests using body heat, measuring its *V* under different activity states. Figure 6f compares the *V* during standing and moving states, with the inset showing a photograph of the wearable device. The series of tests indicate that when the wearer is in motion, the *V* increases rapidly due to the rise in body temperature and the enhanced heat dissipation effect from stronger air convection. When the wearer returns to a standing state, the *V* decreases to normal levels, demonstrating the potential for practical wearable applications.

The performance of the p‐type material is limited by its relatively low *σ*. Future work could focus on improving p‐type material performance through methods such as doping, which is a common strategy for optimizing thermoelectric properties. For Mg_3_Bi_2_ bulk materials, Sb and Te are frequently used as dopants.^[^ ^29^, ^31^ ^]^ Partially substituting Bi with Sb is expected to significantly reduce the *κ*, thus increasing the *ZT* value. Meanwhile, doping with Te can effectively enhance the *n*, which improves the *σ* and optimizes the *S* ^2^ *σ*.

## 3 Conclusion

In this study, we prepare Mg_3_Bi_2_ thin films using magnetron sputtering and ex‐situ annealing and analyze the reaction process of the material in ex‐situ annealing. By controlling the Bi content in the Mg_3_Bi_2_ thin films, we manually achieve p‐type and n‐type transitions to fabricate thermoelectric devices. The material exhibited hole conduction characteristics at *y* = 10% and 20%, making it a p‐type Mg_3_Bi_2_, and electron conduction characteristics at *y* = 38%, 40%, 42%, making it an n‐type Mg_3_Bi_2_. A peak *ZT* of 0.19 at 60 °C is achieved with Bi content 40%. By controlling the thin film thickness and using a flexible polyimide substrate, the thin films maintained a performance change within 10% after 500 bending cycles with a radius of only 5 mm, demonstrating excellent flexibility and high adhesion to the substrate. Based on the as‐fabricated thin films, we modularly fabricated a wearable device with a pair of hot legs, which exhibited a *V* of 0.9 mV, *P* of 0.17 nW, and *ω* of 1.67 µW cm^−2^ at a Δ*T* = 2.5 K. This study introduces a relatively simple Mg_3_Bi_2_ thin film preparation process and demonstrates the feasibility of large‐scale production of thin film thermoelectric materials.

## 4 Experimental Section

The experimental details of this work can be seen in the Supporting Information.

## Conflict of Interest

The authors declare no conflict of interest.

## Supporting information



Supporting Information

## Data Availability

The data that support the findings of this study are available from the corresponding author upon reasonable request.
