# Supplementary material for: High‐Performing Flexible Mg3Bi2 Thin‐Film Thermoelectrics
Source: Adv Sci (Weinh). 2024 Oct 1;11(44):2409788. doi: 10.1002/advs.202409788 (PMC11600257; doi:10.1002/advs.202409788)
Supplement: Supplementary file 1 — Supporting Information [file ADVS-11-2409788-s001.docx]

Supporting Information

**High-performing flexible Mg_3_Bi_2_ thin-film thermoelectrics**

*Boxuan Hu, Xiao-Lei Shi,^*^ Tianyi Cao, Siqi Liu, Min Zhang, Wanyu Lyu, Liangcao Yin, Tuquabo Tesfamichael, Qingfeng Liu, and Zhi-Gang Chen^*^*

B.X. Hu, Dr. X.-L. Shi, T.Y. Cao, S.Q Liu, M Zhang, Dr. W.Y. Lyu, Prof. Z.-G. Chen

School of Chemistry and Physics, ARC Research Hub in Zero-emission Power Generation for Carbon Neutrality, and Centre for Materials Science, Queensland University of Technology, Brisbane, Queensland 4000, Australia

E-mail: xiaolei.shi@qut.edu.au (X.-L. Shi); zhigang.chen@qut.edu.au (Z.-G. Chen)

L.C. Yin, Prof. Q.F. Liu

State Key Laboratory of Materials-Oriented Chemical Engineering, College of Chemical Engineering, Nanjing Tech University, Nanjing 211816, China

Dr. T. Tesfamichael

School of Mechanical, Medical and Process Engineering, Queensland University of Technology, Brisbane, Queensland, 4001 Australia

**Keywords**: thermoelectric, Mg_3_Bi_2_, thin film, flexibility, device.

**1 Experimental Details**

*1. Material synthesis*

Mg_3_Bi_2_ thin films were prepared on polycrystalline Al_2_O_3_ substrate by co-deposition using magnetron sputtering (PVD 75 magnetron sputtering, Kurt J. Lesker). The substrate (15×13×0.5 mm, Maideli) was ultrasonically cleaned with acetone and ethanol for 15 min before the deposition was started. Mg (99.99% purity) and Bi (99.99 purity) targets (dia 50.8×6.35 mm th, Maideli) were used for co-deposition of the thin films. The chamber was evacuated to a base-pressure below 5×10^-6^ Torr The films were deposited at working pressure of 10 mTorr, deposition power between 45-80 W for Mg and 5-15 W for Bi, and deposition duration of 1 h30 mins. For homogeneity of the films, the substrate holder was rotated at 10 rpm. After deposition, the samples were placed in a tube furnace (Across international) and annealed at 265°C under a flow of Ar at 100 ml/min to obtain Mg_3_Bi_2_ film samples for testing.

*1.2. Characterizations*

Grazing incident X-ray diffraction (XRD) analysis was conducted using a Rigaku Smart Lab instrument with CuKα radiation across an angular range of 20° to 70° in 0.02° increments to ascertain the crystal orientation of the Mg_3_Bi_2_ thin-film samples. The Morphological analysis, mapping, and composition assessment of the samples were performed using a Hitachi SU7000 scanning electron microscope (SEM). An energy-dispersive X-ray spectroscopy (EDS) detector from Oxford Ultim Max 100 EDS, was utilized for EDS analysis. Furthermore, lamina samples of Mg_3_Bi_2_ thin films were prepared using the FEI Scios FIB. The spherical aberration-corrected scanning transmission electron microscopy (Cs-TEM) (Jeol NEOARM) was employed for high-angle annular dark-field imaging (HAADF) in scanning TEM (STEM) mode to conduct microanalysis of Mg_3_Bi_2_ thin film FIB samples. Film thickness measured by stylus profiler (Bruker Dektak stylus profiler). A mold was used to cover a portion of the substrate during film deposition, ensuring that only part of the substrate was coated with the film. After deposition, the film was annealed, and the difference in surface height between the areas with and without Mg_3_Bi_2_ was measured using a stylus profiler. This measurement provided the thickness of the deposited material.

*1.3. Thermoelectric performance evaluation*

The electrical conductivity σ and Seebeck coefficient S were measured using a Seebeck coefficient and electrical conductivity apparatus (ZEM-3). Thermal conductivity κ was determined using the alternative current method thermal diffusivity D measurement system (RIKO Laser-PIT). Carrier concentration n and mobility μ were investigated using a Van der Pauw Hall measuring instrument (CH-70, CH-magnetoelectricity Technology Co., Ltd., China) under a magnetic field up to 500 mT. n and μ were determined by 𝑛 = 1⁄𝑒𝑅 and 𝜇 = 𝜎𝑅, respectively.

*1.4. Device assembly and performance evaluation*

The design of the device was modularized. First, silver electrodes and silver conductors were deposited on PI films using electron beam evaporation and mask techniques. Subsequently, p-type and n-type Mg_3_Bi_2_ films were co-deposited on Al_2_O_3_ using magnetron sputtering and annealed at 265°C for 15 h. Then, the back side of the Mg_3_Bi_2_ film (Al_2_O_3_ side) was adhered to the PI by double-sided adhesive, and the hot and cold sides of the material connected to the electrodes by using a thermosetting silver gel, which was inserted into a suitably tailored silicone gel base when it was thermoset. This created a modular device. After assembly, thermal gradient simulations were performed using a hot plate stirrer (IKA C-MAG HS7) to evaluate the thermoelectric properties. Under load application, the output performance was measured using a multimeter/DC power supply unit (KEYSIGHT U3606B) and a regular multimeter. A regular multimeter was used to measure the load voltage, while the multimeter/DC power supply unit was used to measure the test current.

*1.5. Single parabolic (SPB) modelling*

We employed an SPB model to perform a simulation to understand our transport properties:^[1-4]^

$S\left( \eta\right)=\frac{k_{B}}{e}.\left[ \frac{\left( r+\frac{5}{2} \right).F_{r+\frac{3}{2}}\left( \eta\right)}{\left( r+\frac{3}{2} \right).F_{r+\frac{1}{2}}\left( \eta\right)}-\eta\right]$ (S-01)

$n_{H}=\frac{1}{e.R_{H}}=\frac{\left( 2m^{*}.k_{B}T \right)^{\frac{3}{2}}}{3\pi^{2}\hbar^{3}}.\frac{\left( r+\frac{3}{2} \right)^{2}.{F_{r+\frac{1}{2}}}^{2}\left( \eta\right)}{\left( 2r+\frac{3}{2} \right).F_{2r+\frac{1}{2}}\left( \eta\right)}$ (S-02)

$\mu_{H}=\left[ \frac{e\pi\hbar^{4}}{\sqrt{2}\left( k_{B}T \right)^{\frac{3}{2}}}\frac{C_{1}}{E_{def}^{2}\left( m^{*} \right)^{\frac{5}{2}}} \right]\frac{\left( 2r+\frac{3}{2} \right).F_{2r+\frac{1}{2}}\left( \eta\right)}{\left( r+\frac{3}{2} \right)^{2}.F_{r+\frac{1}{2}}\left( \eta\right)}$ (S-03)

where $\eta$, $k_{B}$, $e$, $r$, $R_{H}$, $m^{*}$, $\hbar$, $C_{1}$ and $E_{def}$ are the reduced Fermi level, the Boltzmann constant, the electron charge, the carrier scattering factor (*r* = −1/2 for acoustic phonon scattering),^[2]^ the Hall coefficient, the effective mass, the reduced Plank constant, the elastic constant for longitudinal vibrations and the deformation potential coefficient, respectively. Here:

$C_{1}=v_{1}^{2}.\rho$ (S-04)

where $v_{1}$ is the longitudinal sound velocity. $F_{i}\left( \eta\right)$ is the Fermi integral and can be expressed as:

$F_{i}\left( \eta\right)=\int_{0}^{\infty} \frac{x^{i}}{1+e^{\left( x-\eta\right)}}dx$ (S-05)

**2. Supplementary figures**

**
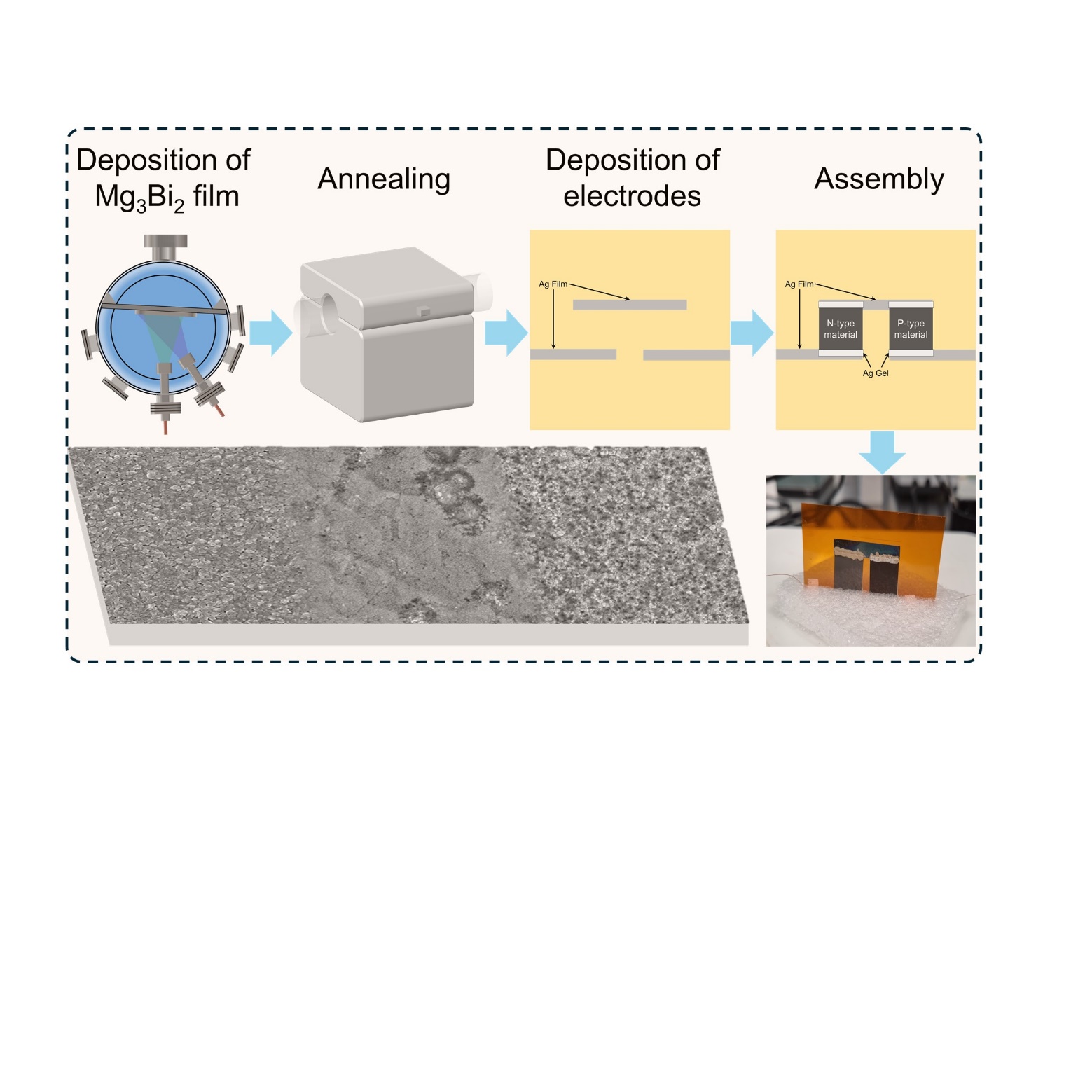
**

**Figure S1.** Illustration of the fabrication process of Mg_3_Bi_2_ thin films and devices.


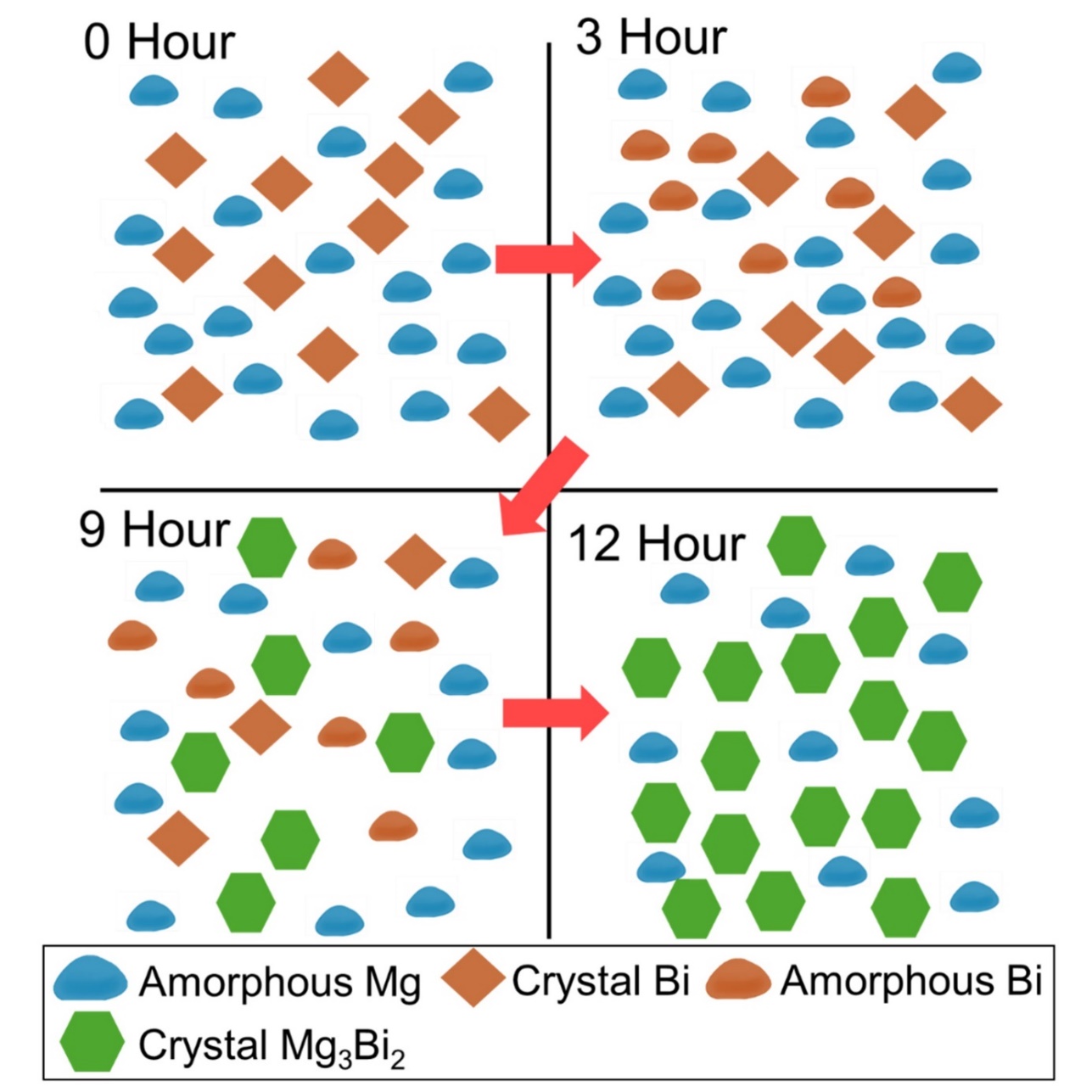


**Figure S2.** Changes of Mg, Bi, and Mg_3_Bi_2_ in films during the annealing process.


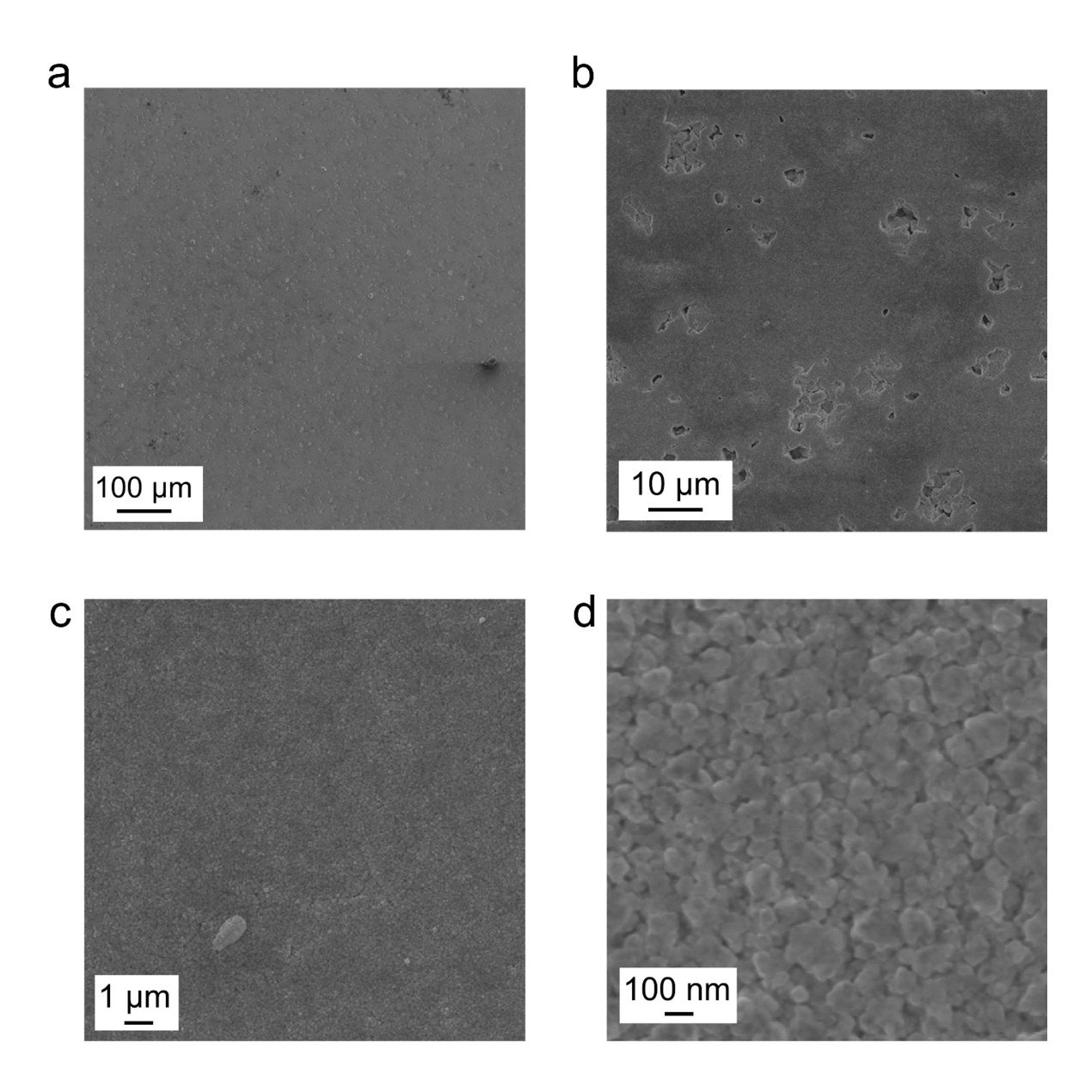


**Figure S3.** Scanning electron microscopy (SEM) image of Mg_3_Bi_2_ film annealed for 3h. **a)** 100, **b)** 1000, **c)** 5000, and **d)** 50000 times.


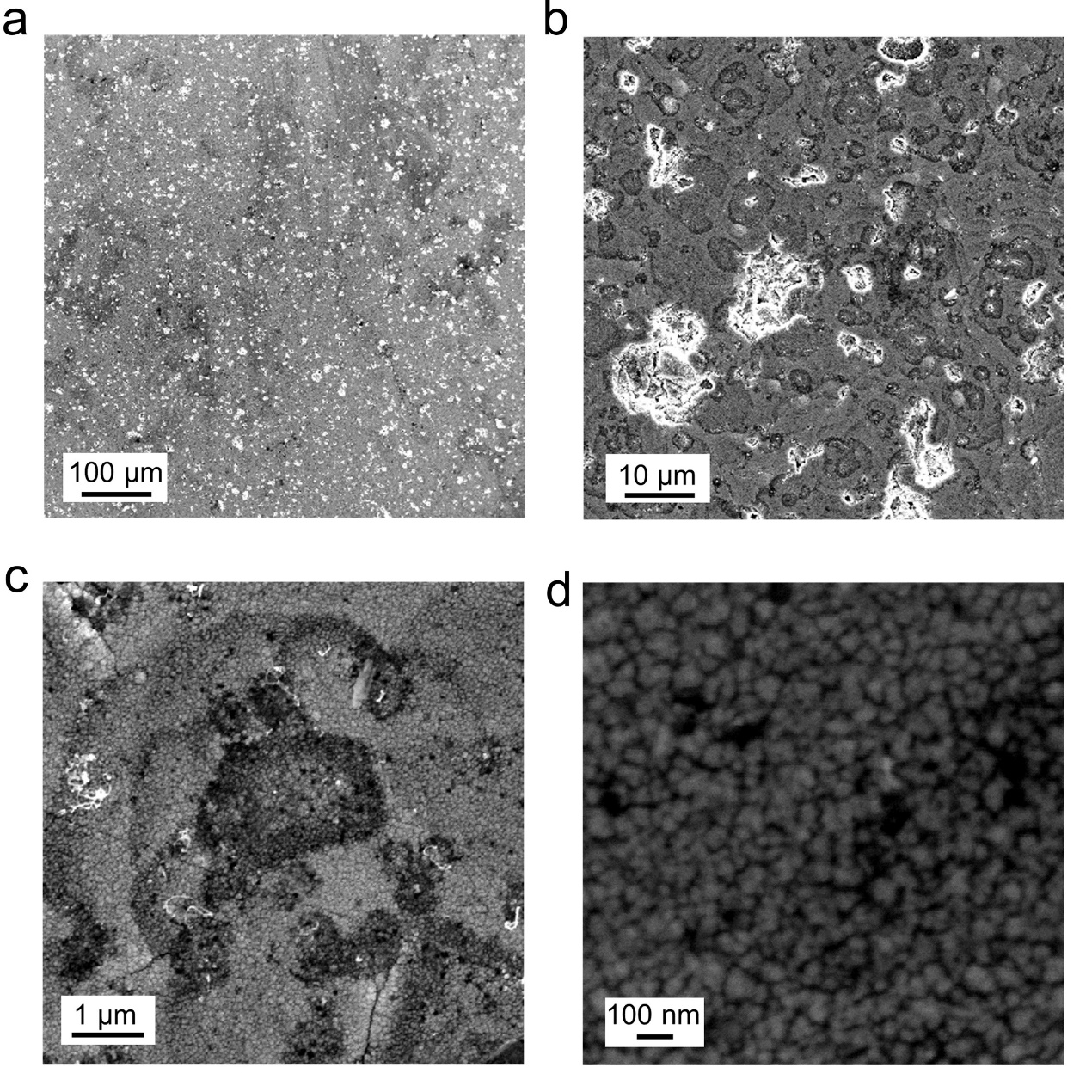


**Figure S4.** SEM image of Mg_3_Bi_2_ film annealed for 6h. **a)** 100, **b)** 1000, **c)** 10000, and **d)** 50000 times.


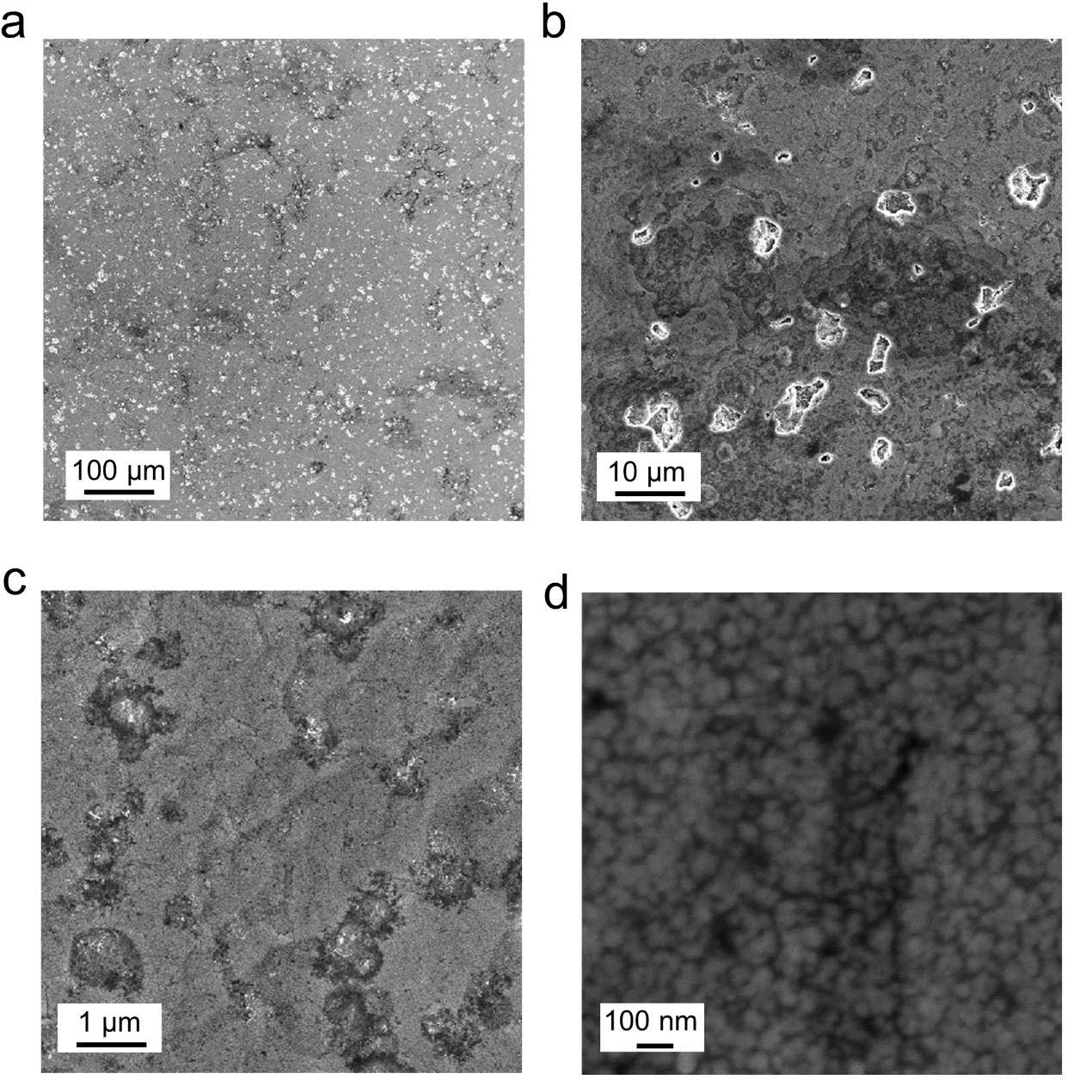


**Figure S5.** SEM image of Mg_3_Bi_2_ film annealed for 9h. **a)** 100, **b)** 1000, **c)** 5000, and **d)** 50000 times.


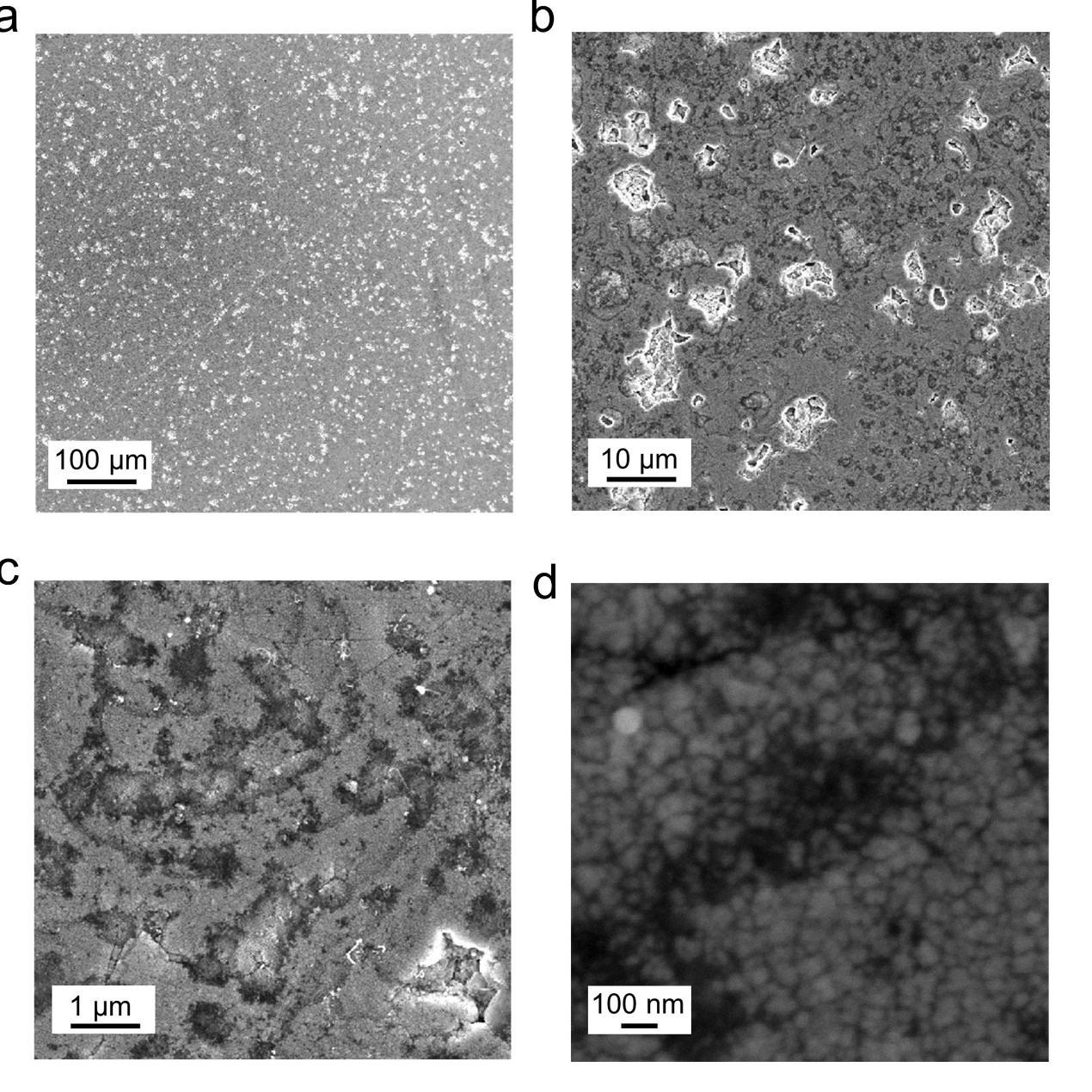


**Figure S6.** SEM image of Mg_3_Bi_2_ film annealed for 12h. **a)** 100, **b)** 1000, **c)** 5000, and **d)** 20000 times.


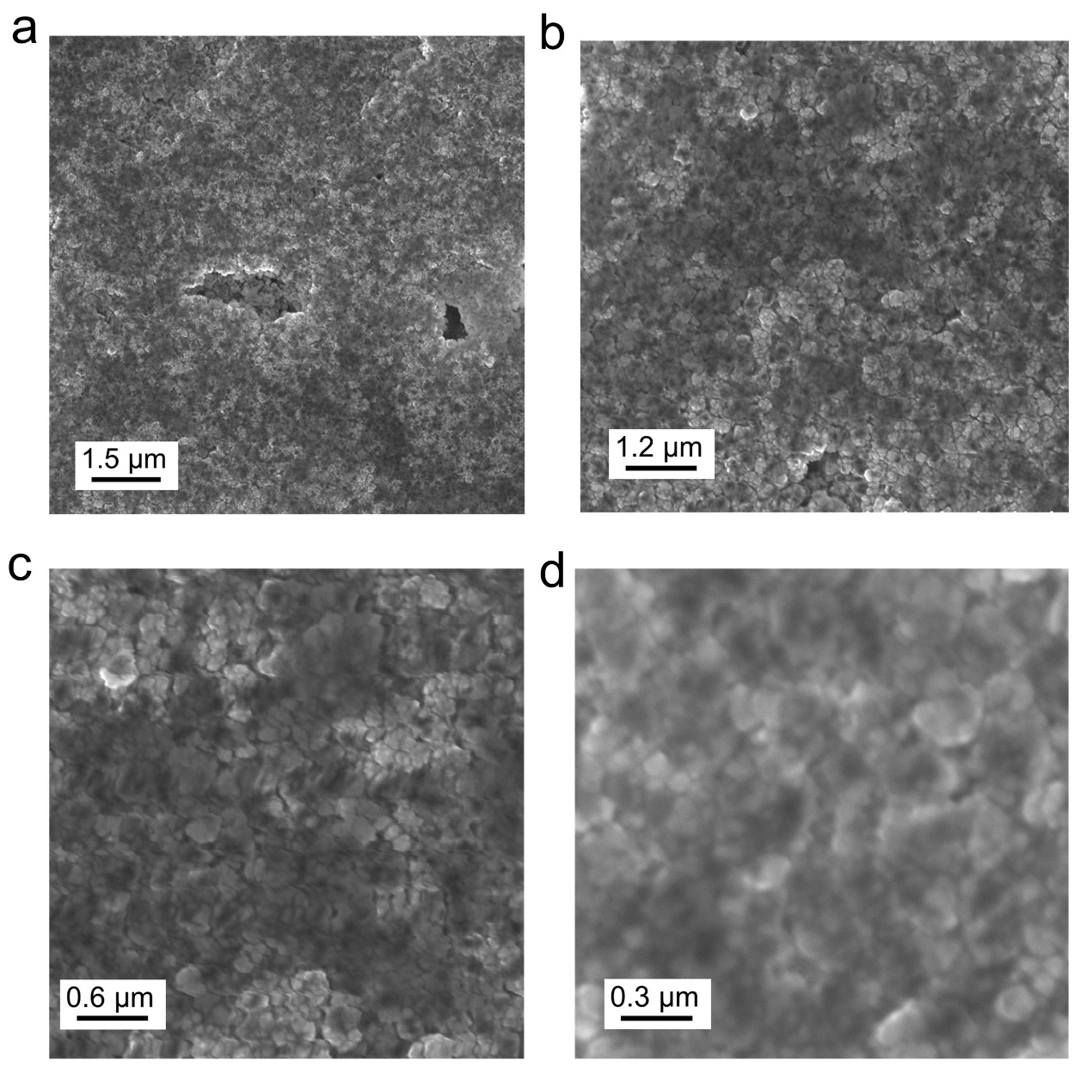


**Figure S7.** SEM image of Mg_3_Bi_2_ (10%Bi). **a)** 6000, **b)** 12000, **c)** 25000, and **d)** 50000 times.


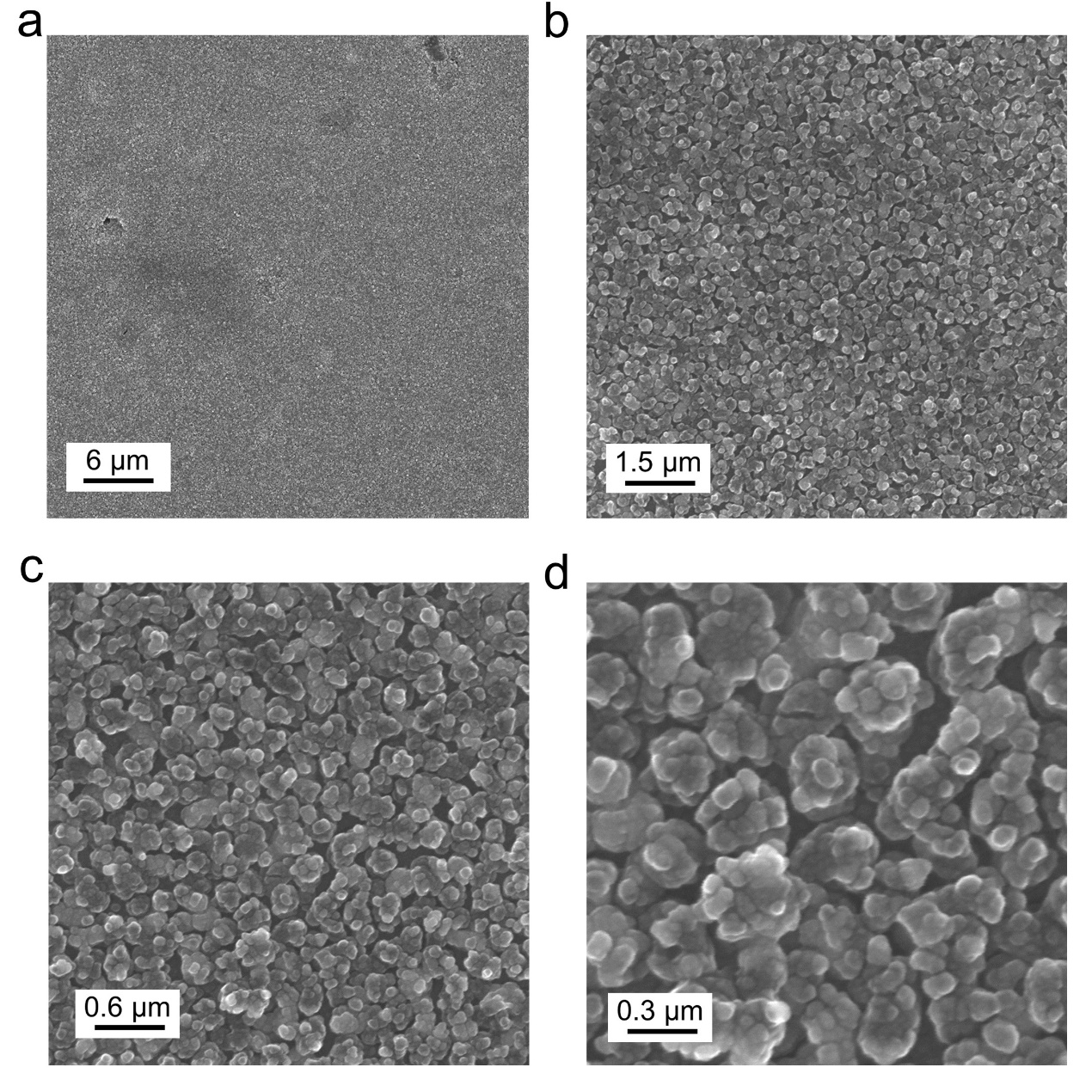


**Figure S8.** SEM image of Mg_3_Bi_2_ (20%Bi). **a)** 2000, **b)** 10000, **c)** 20000, and **d)** 50000 times.


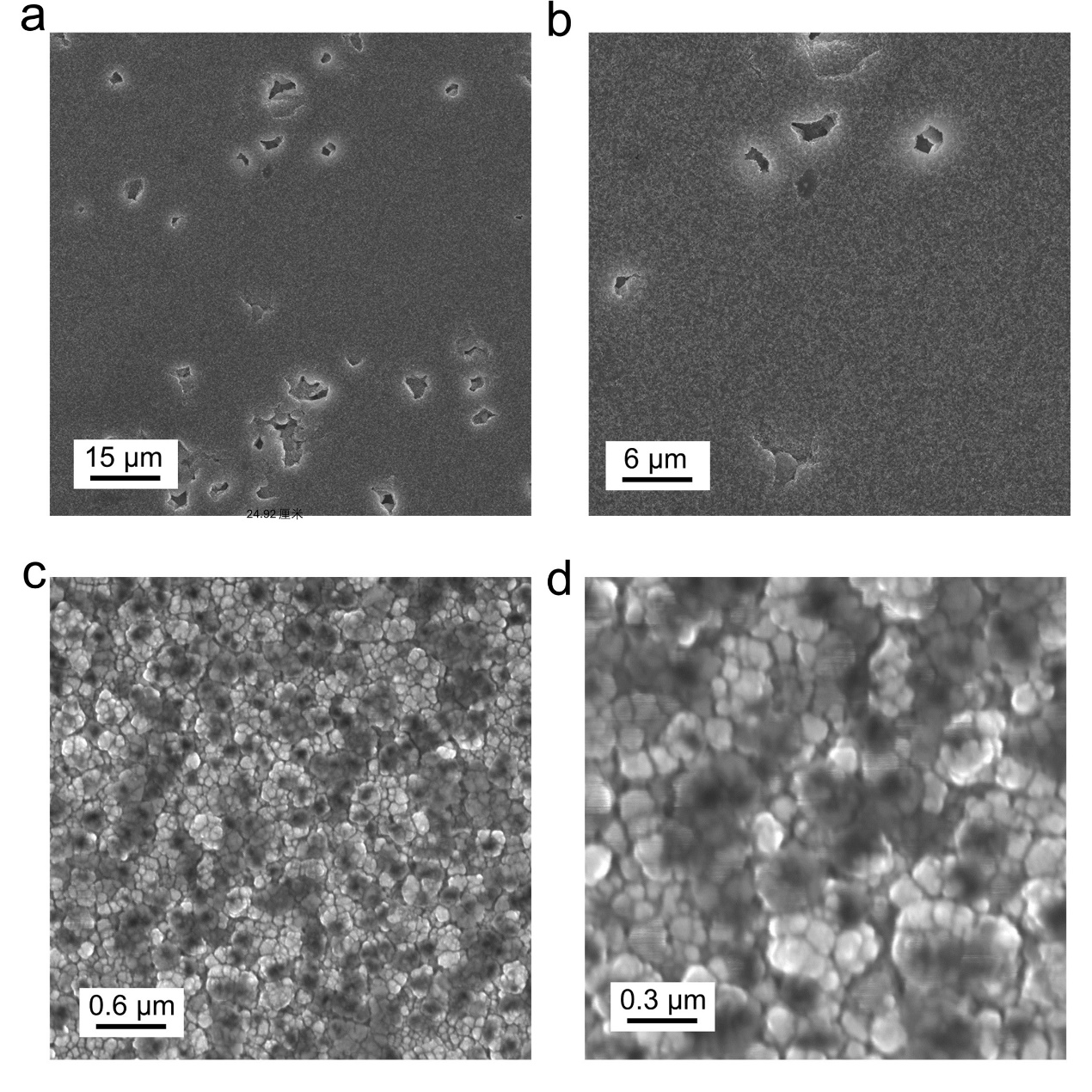


**Figure S9.** SEM image of Mg_3_Bi_2_ (28%Bi). **a)** 1000, **b)** 2000, **c)** 25000, and **d)** 50000 times.


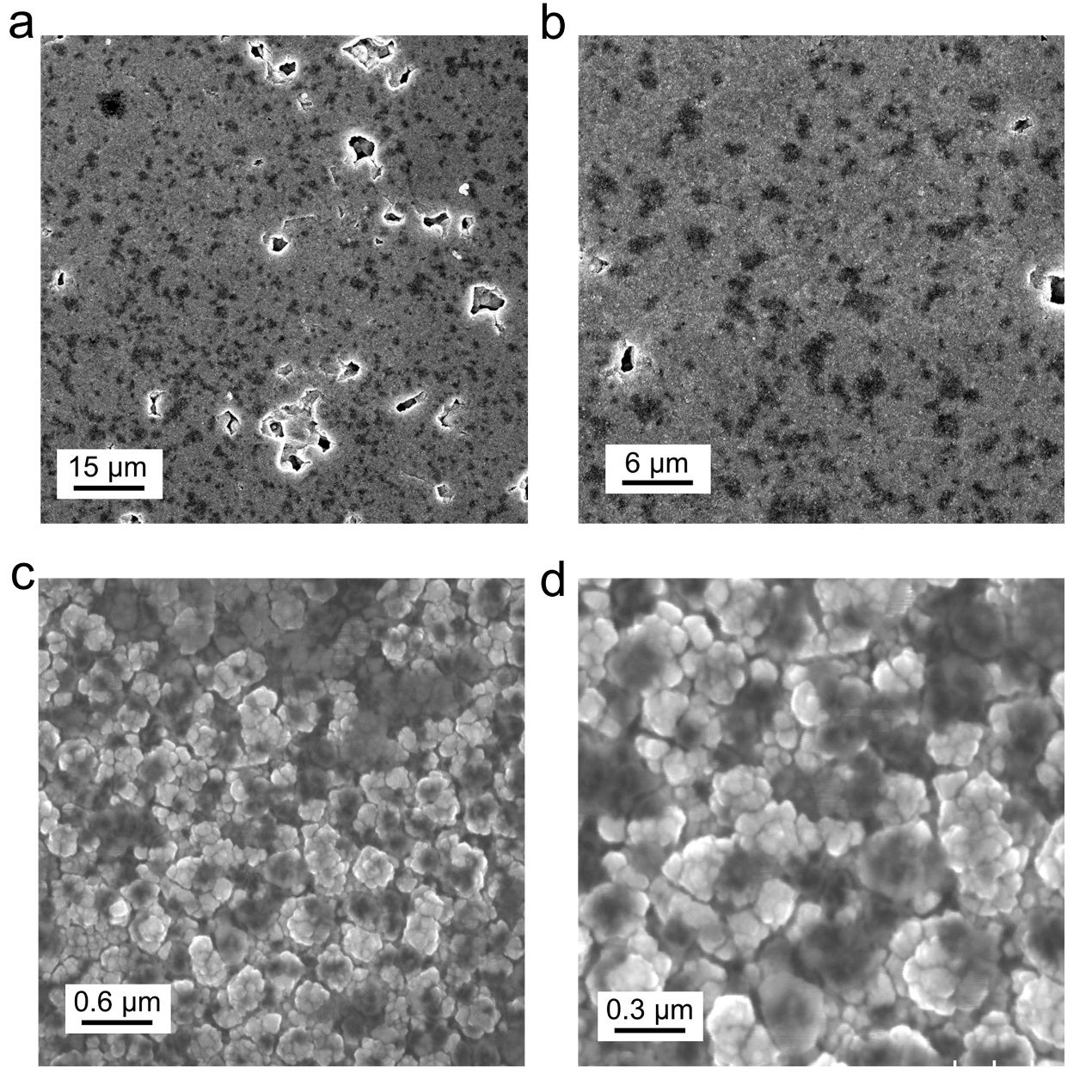


**Figure S10.** SEM image of Mg_3_Bi_2_ (38%Bi). **a)** 1000, **b)** 2000, **c)** 25000, and **d)** 50000 times.


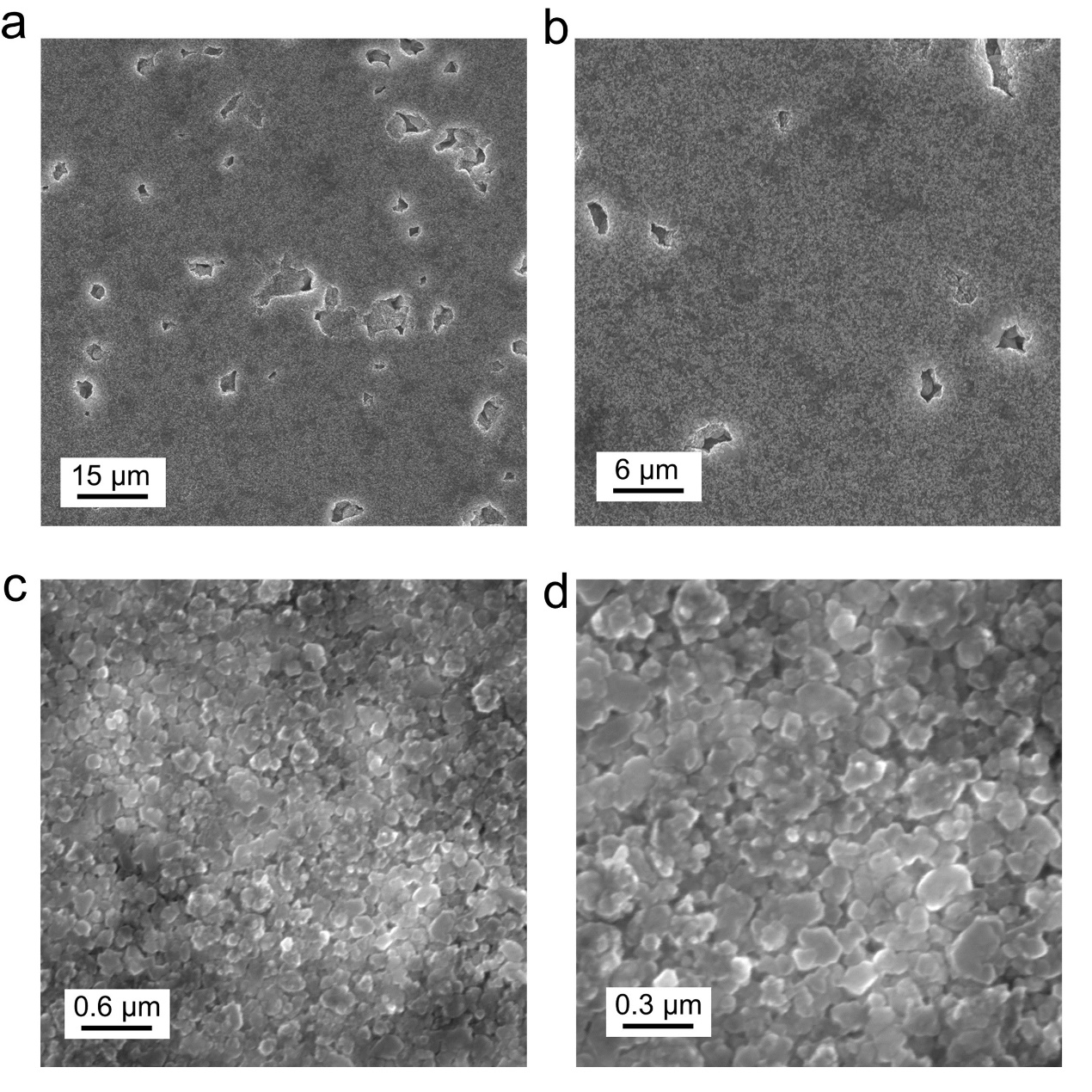


**Figure S11.** SEM image of Mg_3_Bi_2_ (40%Bi). **a)** 1000, **b)** 2000, **c)** 25000, and **d)** 50000 times.

**
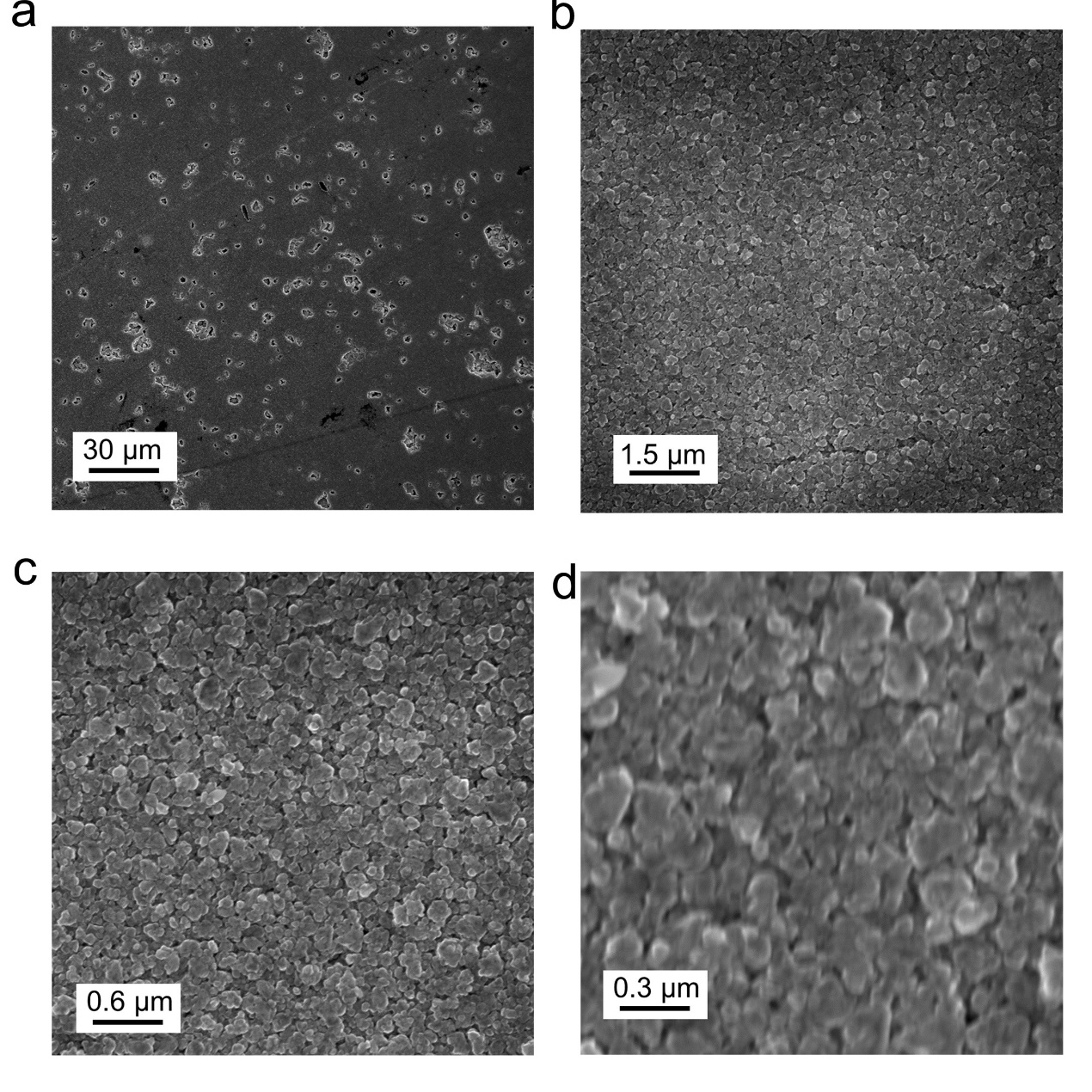
**

**Figure S12.** SEM image of Mg_3_Bi_2_ (42%Bi). **a)** 300, **b)** 10000, **c)** 20000, and **d)** 50000 times.


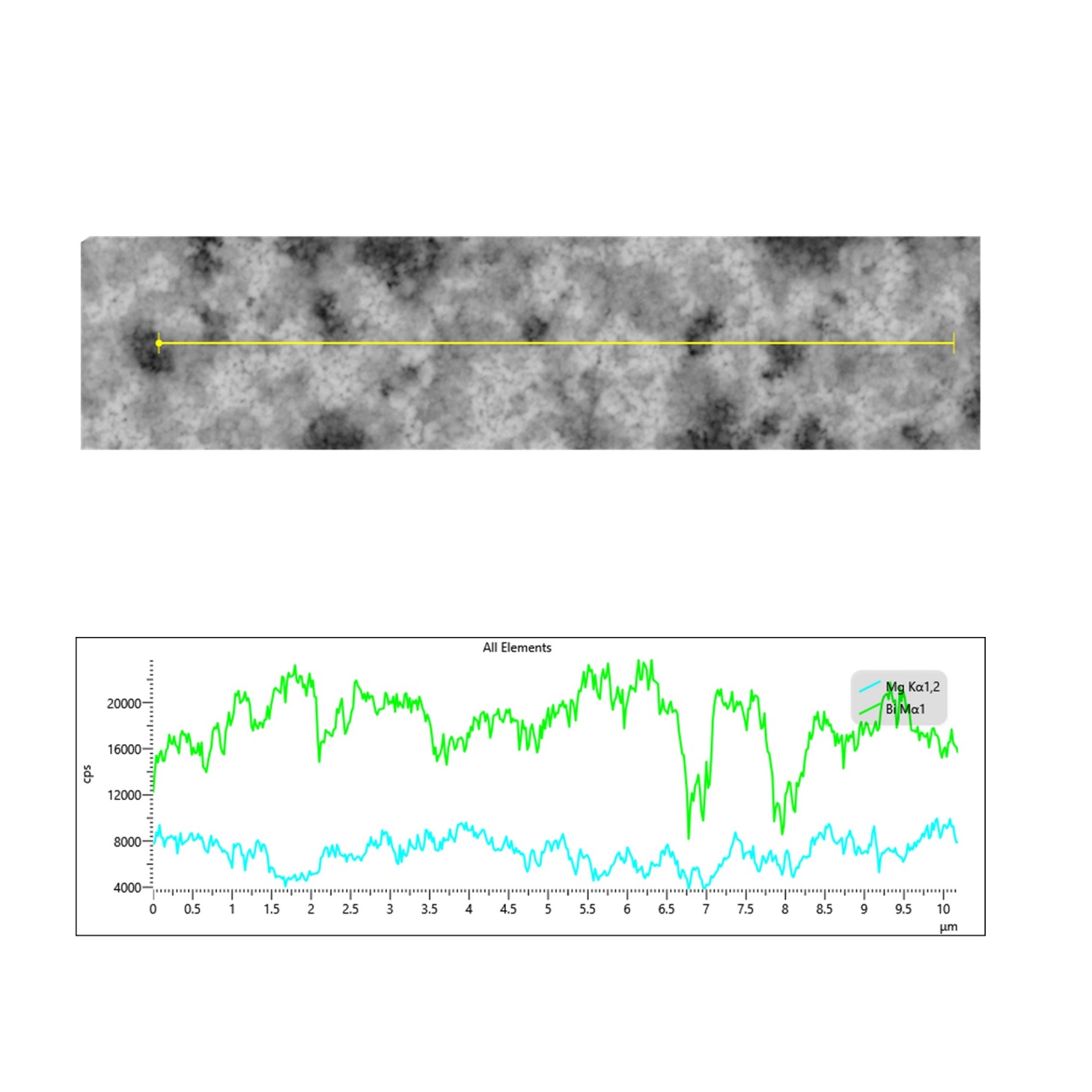


**Figure S13.** Energy-dispersive X-ray spectroscopy (EDS) line-scan image of Mg_3_Bi_2_ (10% Bi), distribution of Mg and Bi.


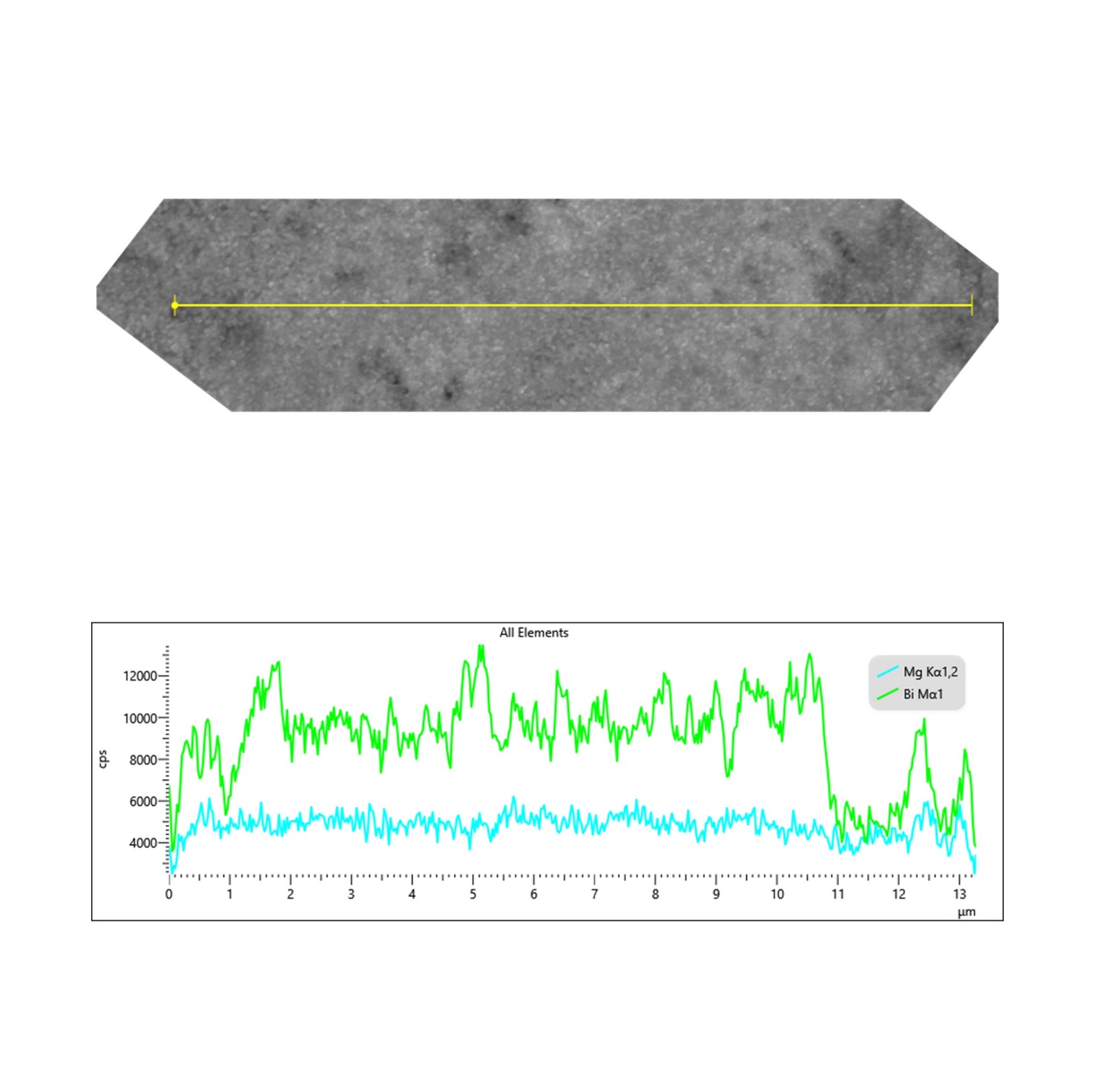


**Figure S14.** EDS line-scan image of Mg_3_Bi_2_ (20% Bi), distribution of Mg and Bi.

**
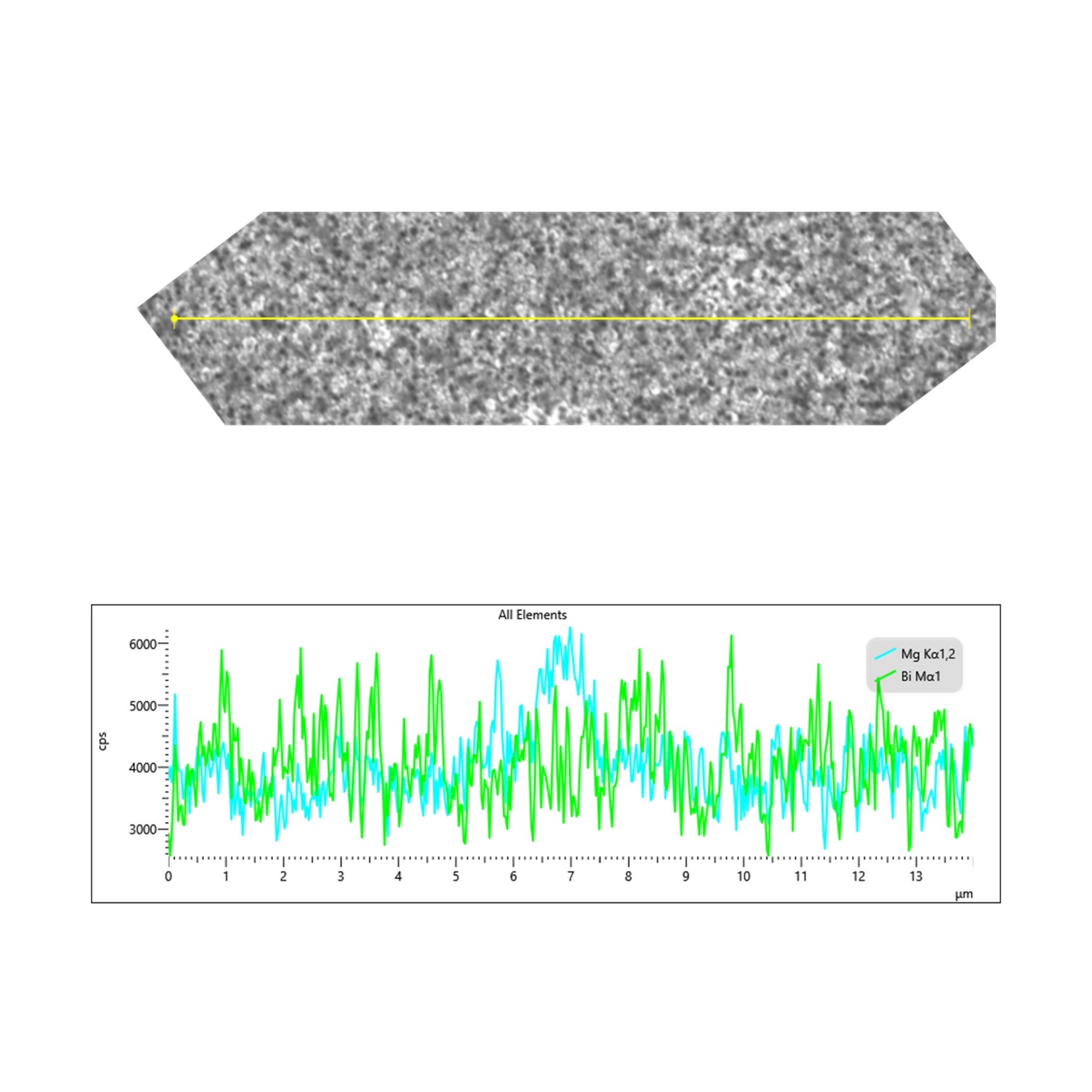
**

**Figure S15.** EDS line-scan image of Mg_3_Bi_2_ (28% Bi), distribution of Mg and Bi.


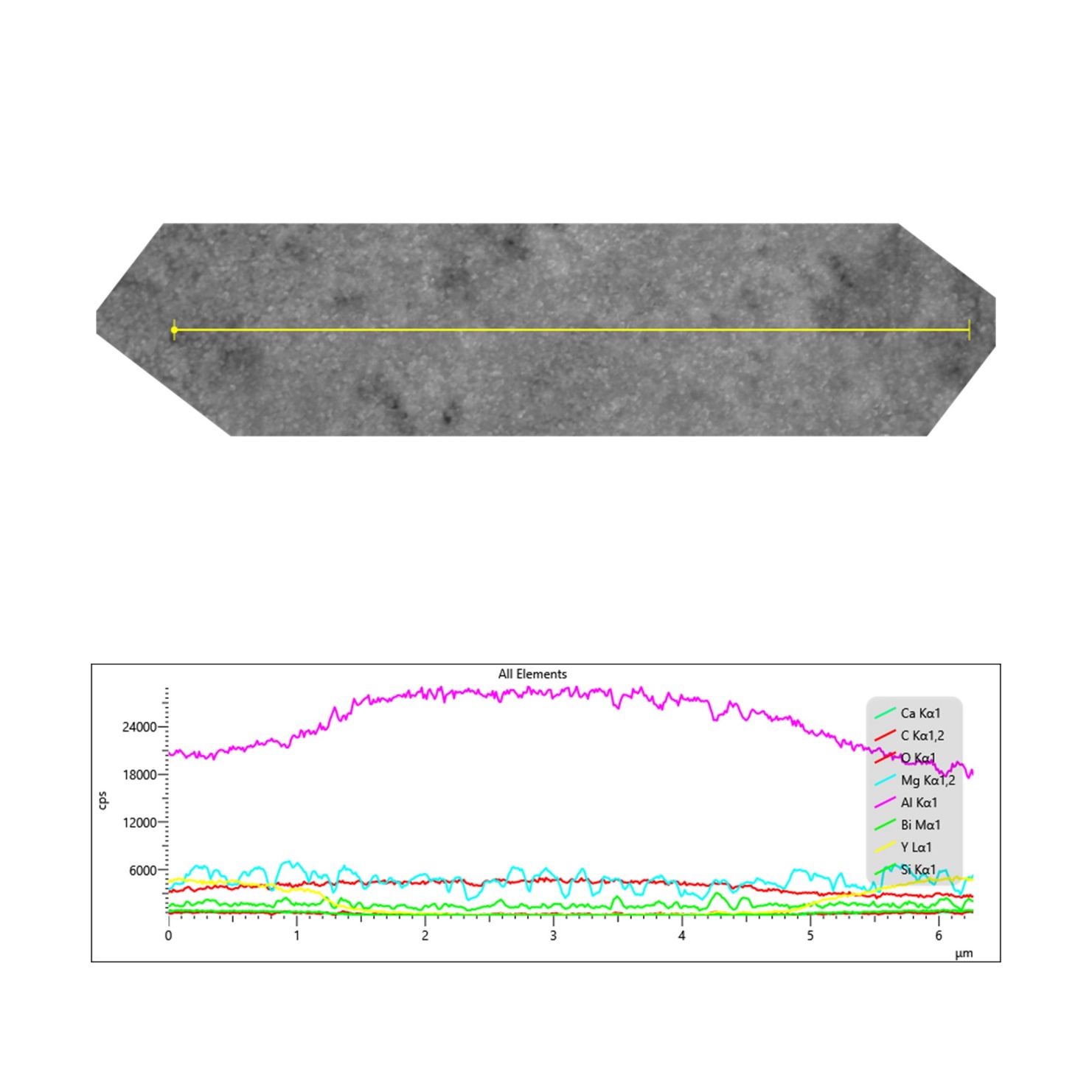


**Figure S16.** EDS line-scan image of Mg_3_Bi_2_ (38% Bi), distribution of Mg and Bi.


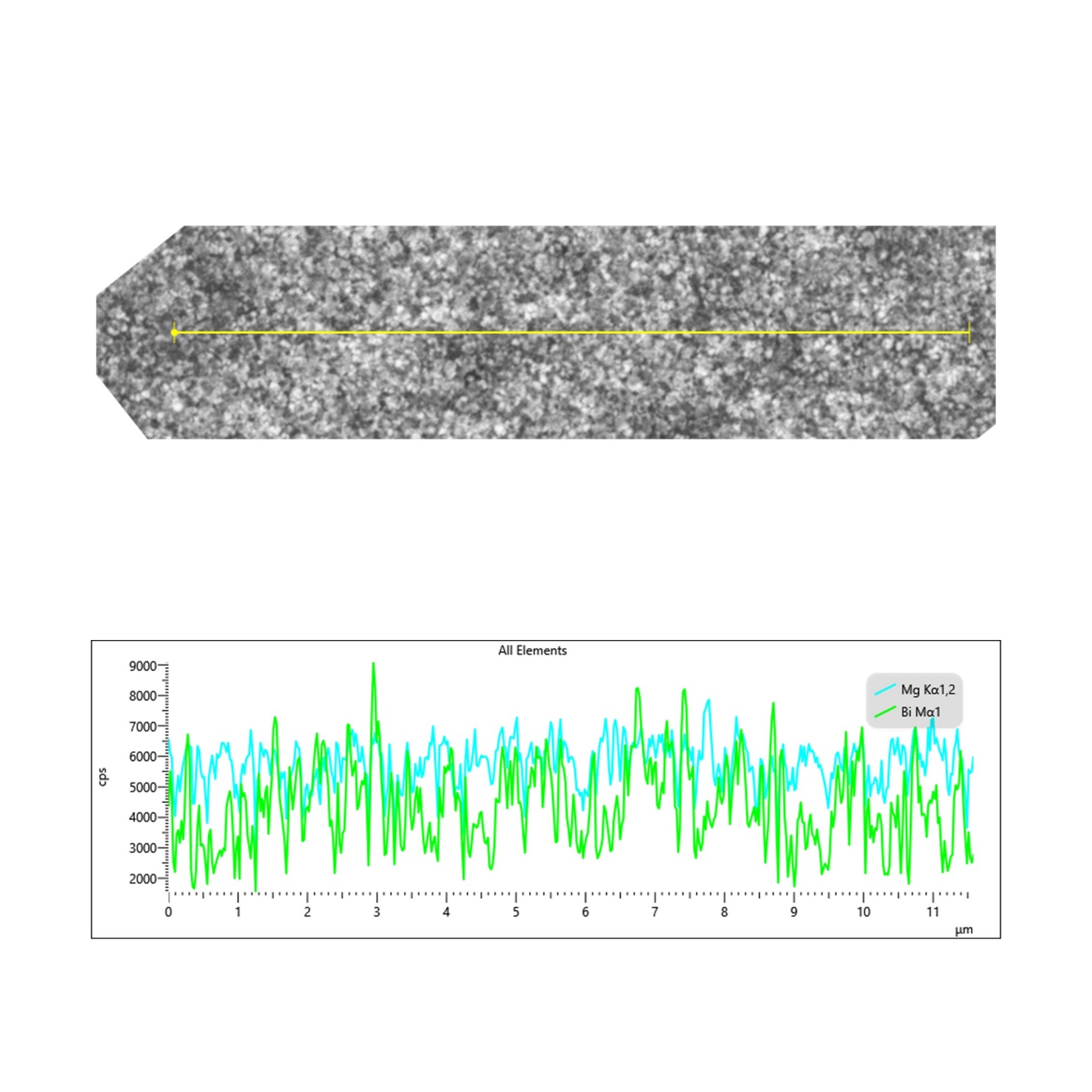


**Figure S17.** EDS line-scan image of Mg_3_Bi_2_ (40% Bi), distribution of Mg and Bi.

**
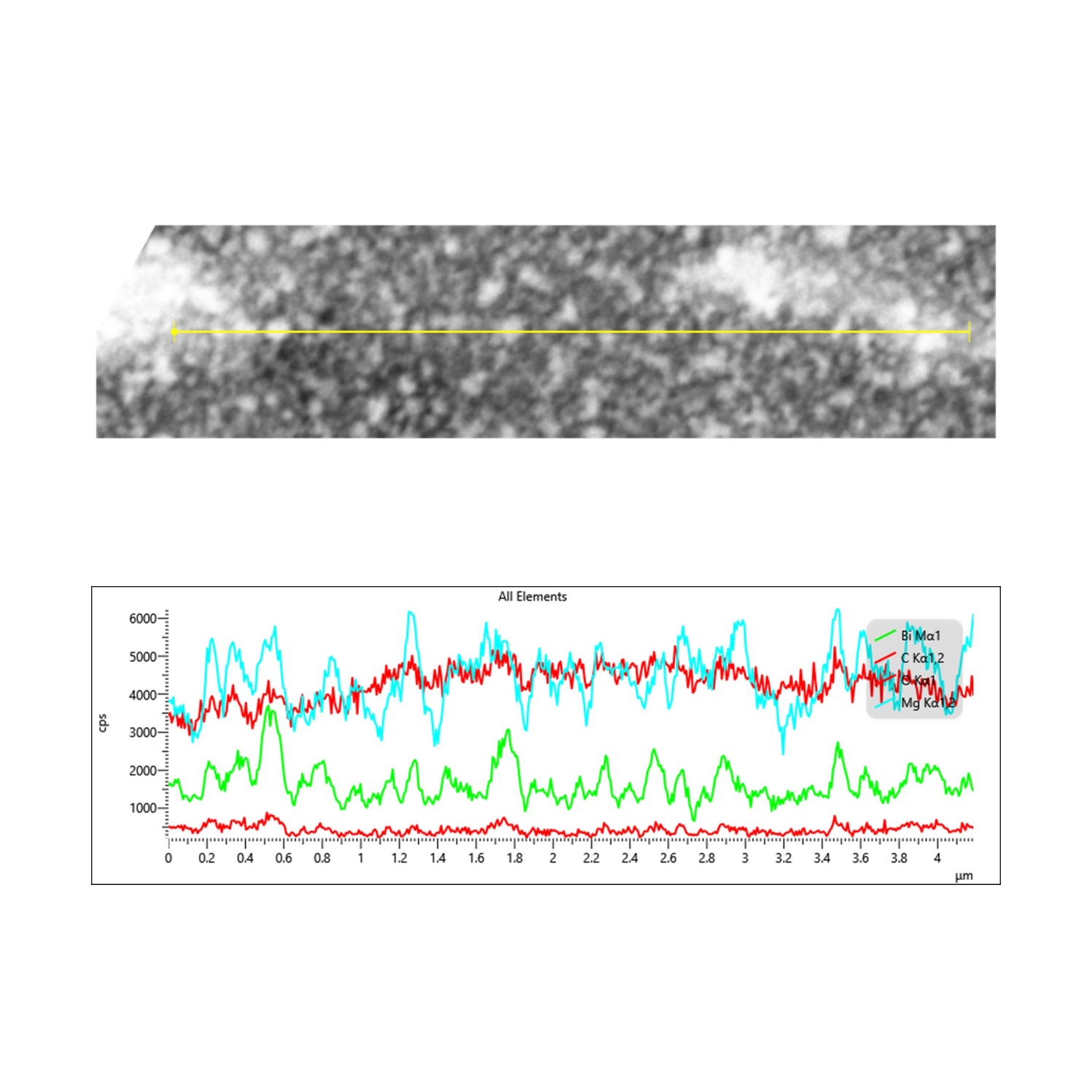
**

**Figure S18.** EDS line-scan image of Mg_3_Bi_2_ (42% Bi), distribution of Mg and Bi.


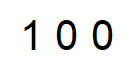
**
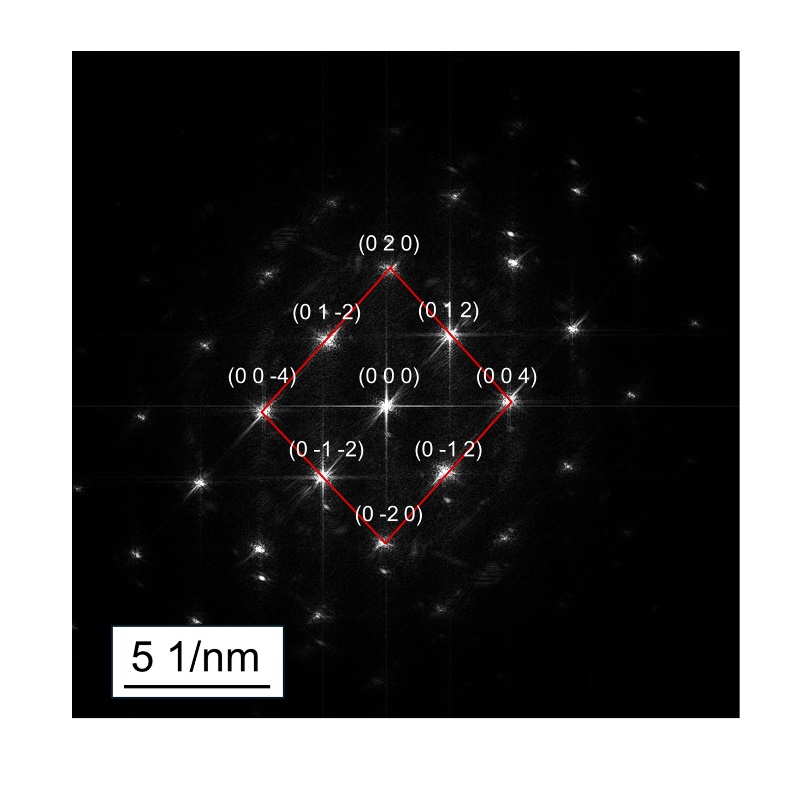
**

**Figure S19.** The corresponding fast Fourier transform (FFT) plot in **Figure 4a**.

**
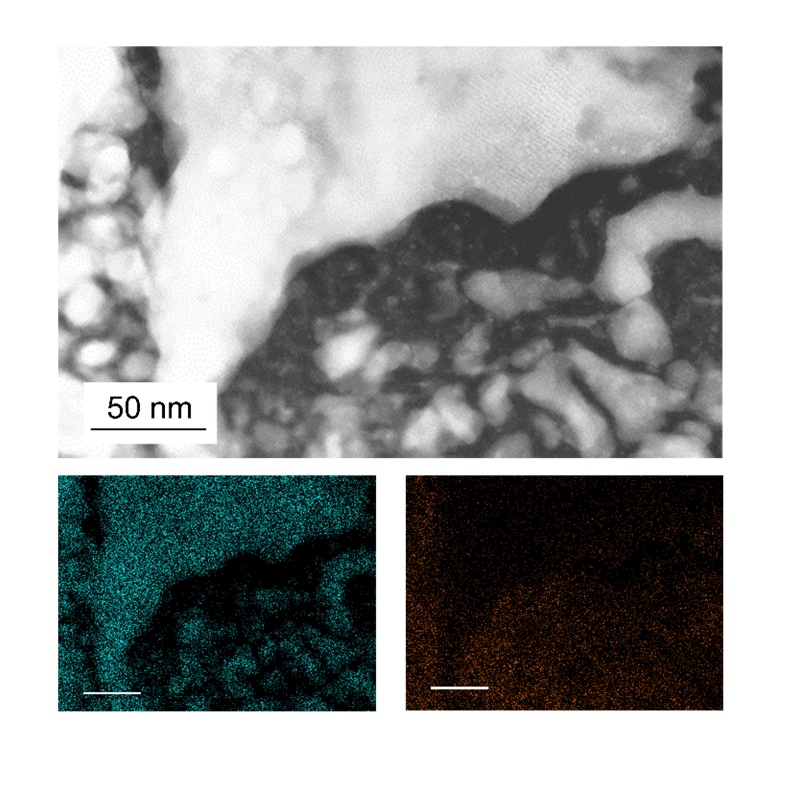
**

**Figure S20.** The overall low-magnification EDS information of **figure 4a**.

**
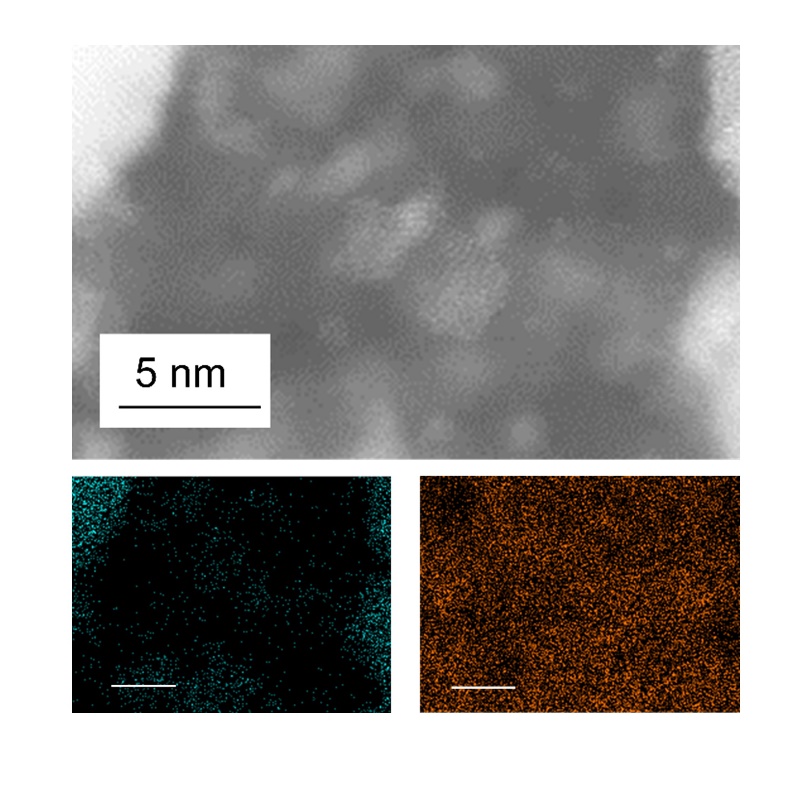
**

**Figure S21.** The amorphous Mg regions EDS information from **figure 4a**.

**
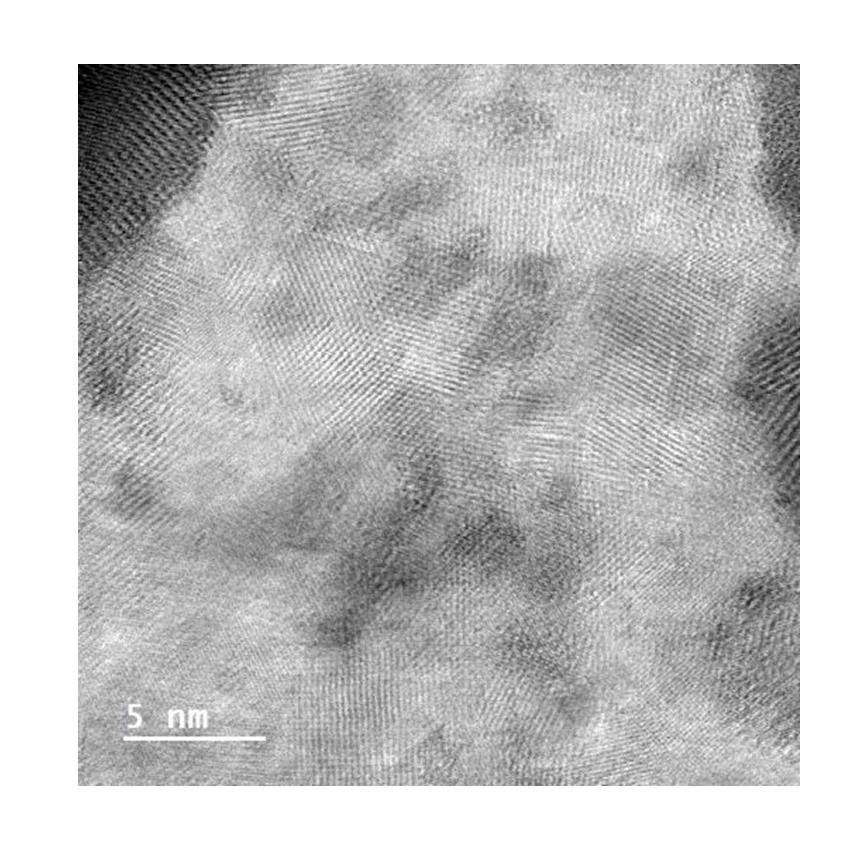
**

**Figure S22.** High-resolution transmission electron microscopy (HRTEM) image of an amorphous Mg region showing the presence of coated Mg_3_Bi_2_ crystals within Mg.

**
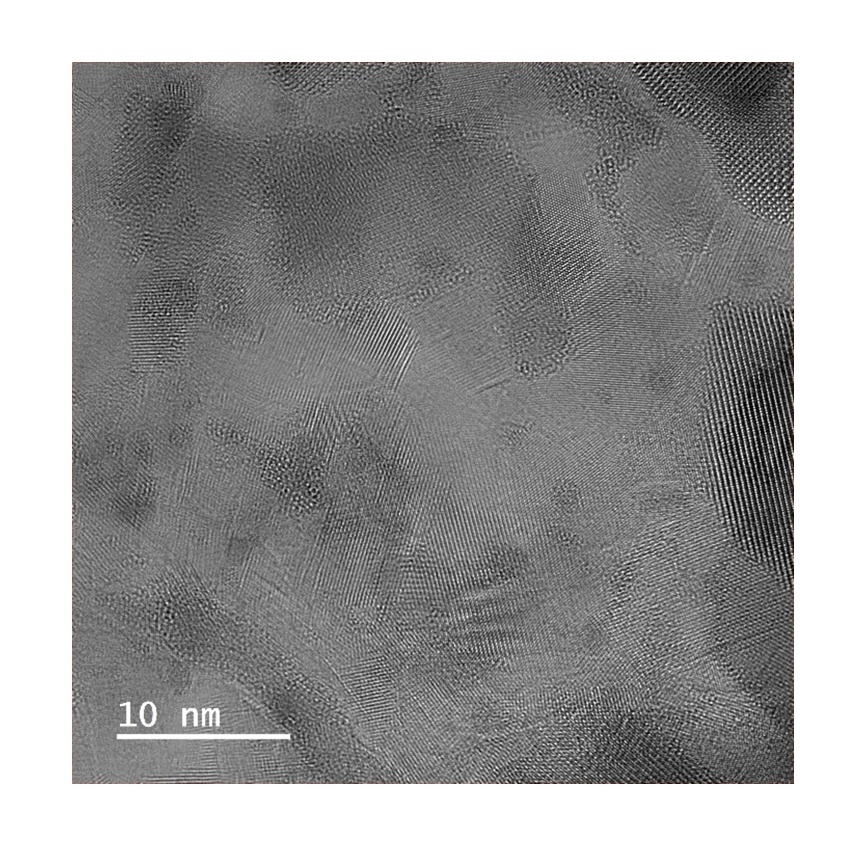
**

**Figure S23.** HRTEM map of the Mg_3_Bi_2_ region showing internal defects.

**
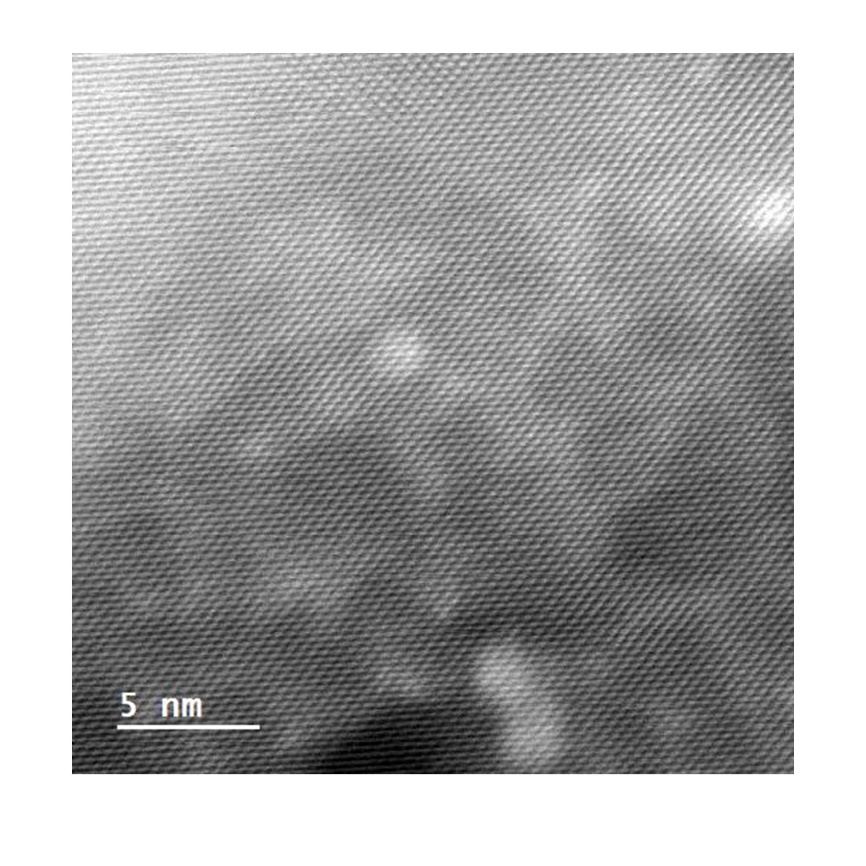
**

**Figure S24.** HRTEM image of the Mg_3_Bi_2_ region showing internal defects.

**
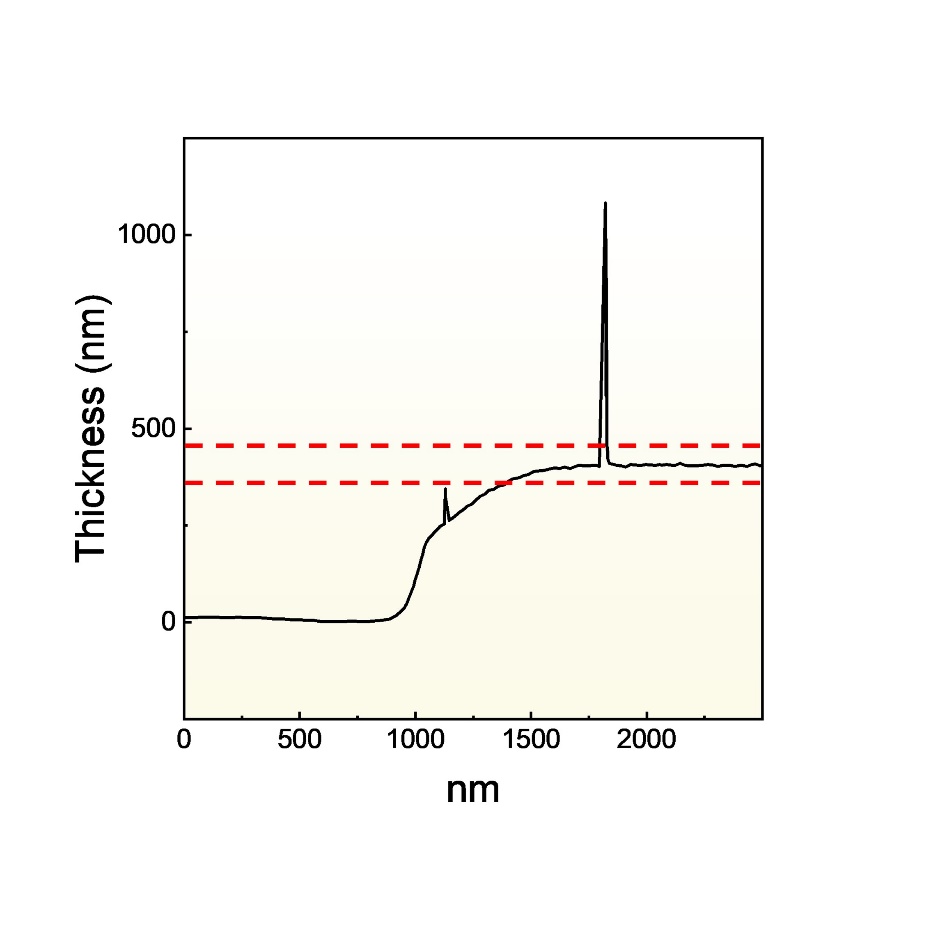
**

**Figure S25.** Thickness measurement of films with 10% Bi content.

**
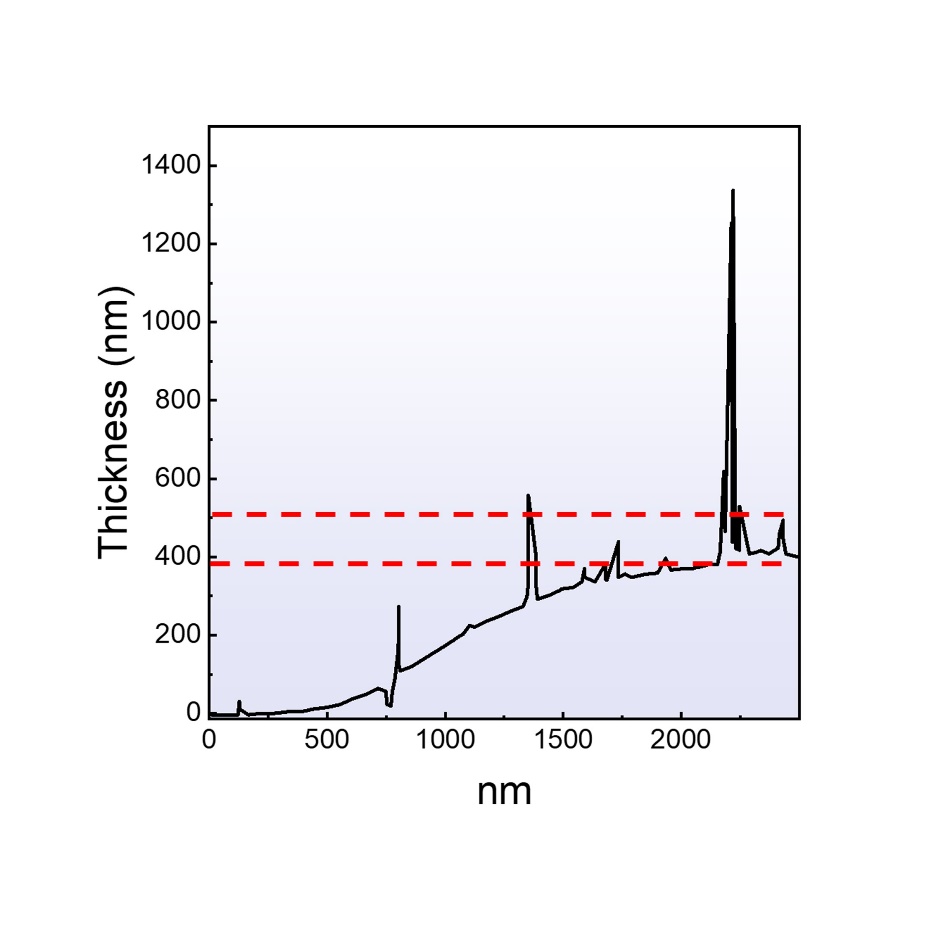
**

**Figure S26.** Thickness measurement of films with 28% Bi content.

**
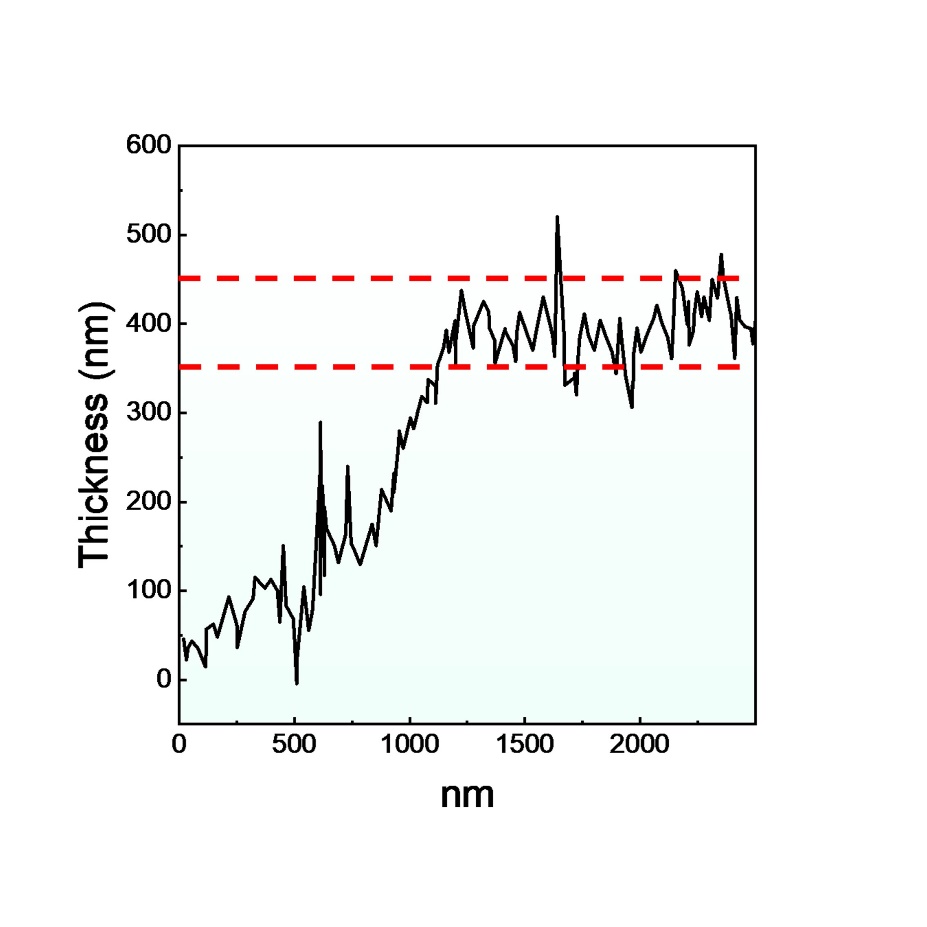
**

**Figure S27.** Thickness measurement of films with 40% Bi content.

**
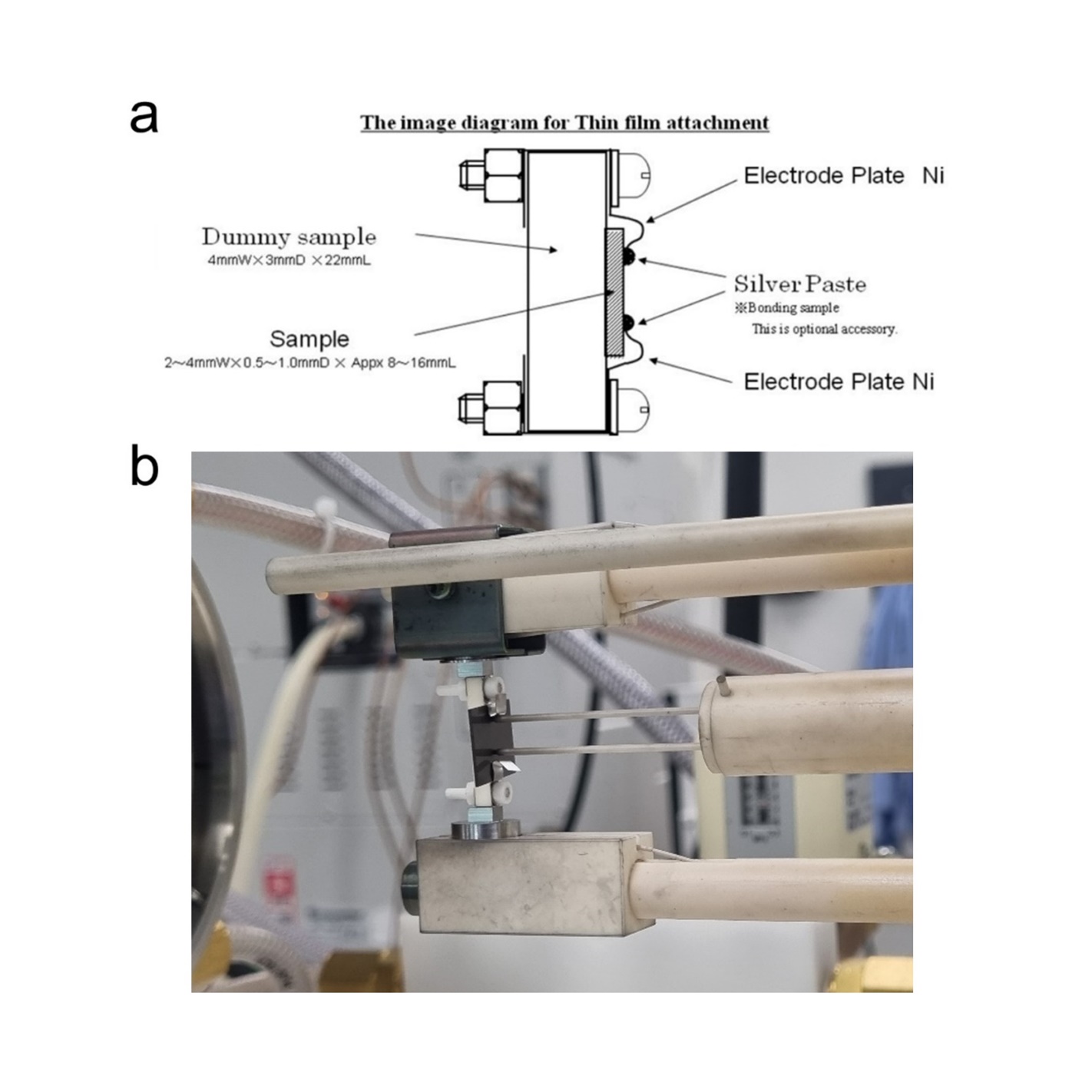
**

**Figure S28.** Measurement of Seebeck coefficient (*S*) and electrical conductivity (*σ*) by employing ZEM 3 for a) structure of sample holder (see <https://ulvac.eu/wp-content/uploads/2022/03/Thermoelectric-Evaluation-Seebeck-Coeeficient-Eletcric-Resistance-Measurement-ZEM-3-series.pdf>) and b) photograph of the testing set-up.


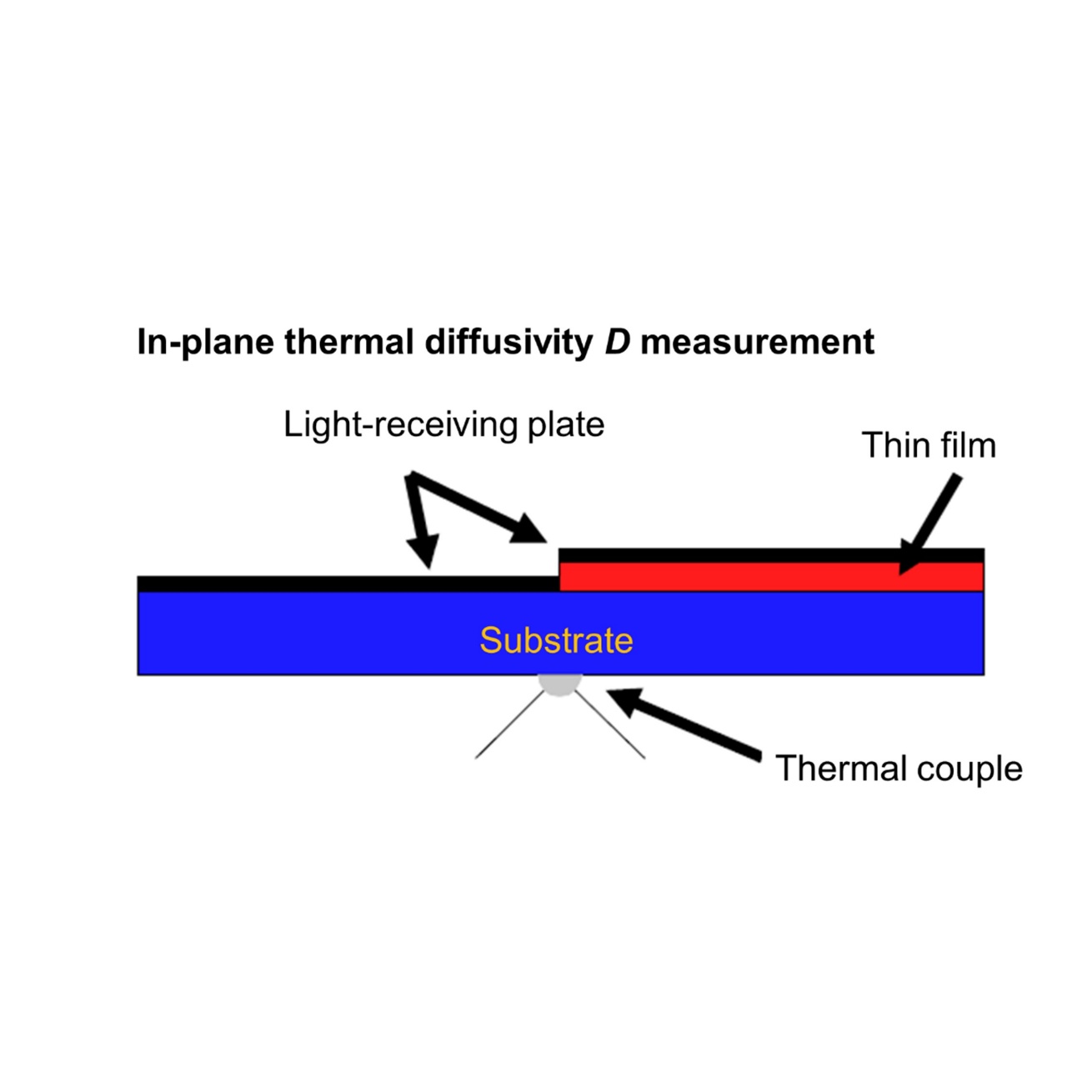


**Figure S29.** Illustration of in-plane thermal diffusivity thermal diffusivity (*D*) measurement using Laser PIT alternative current (AC) method thermal diffusivity measurement system.

**
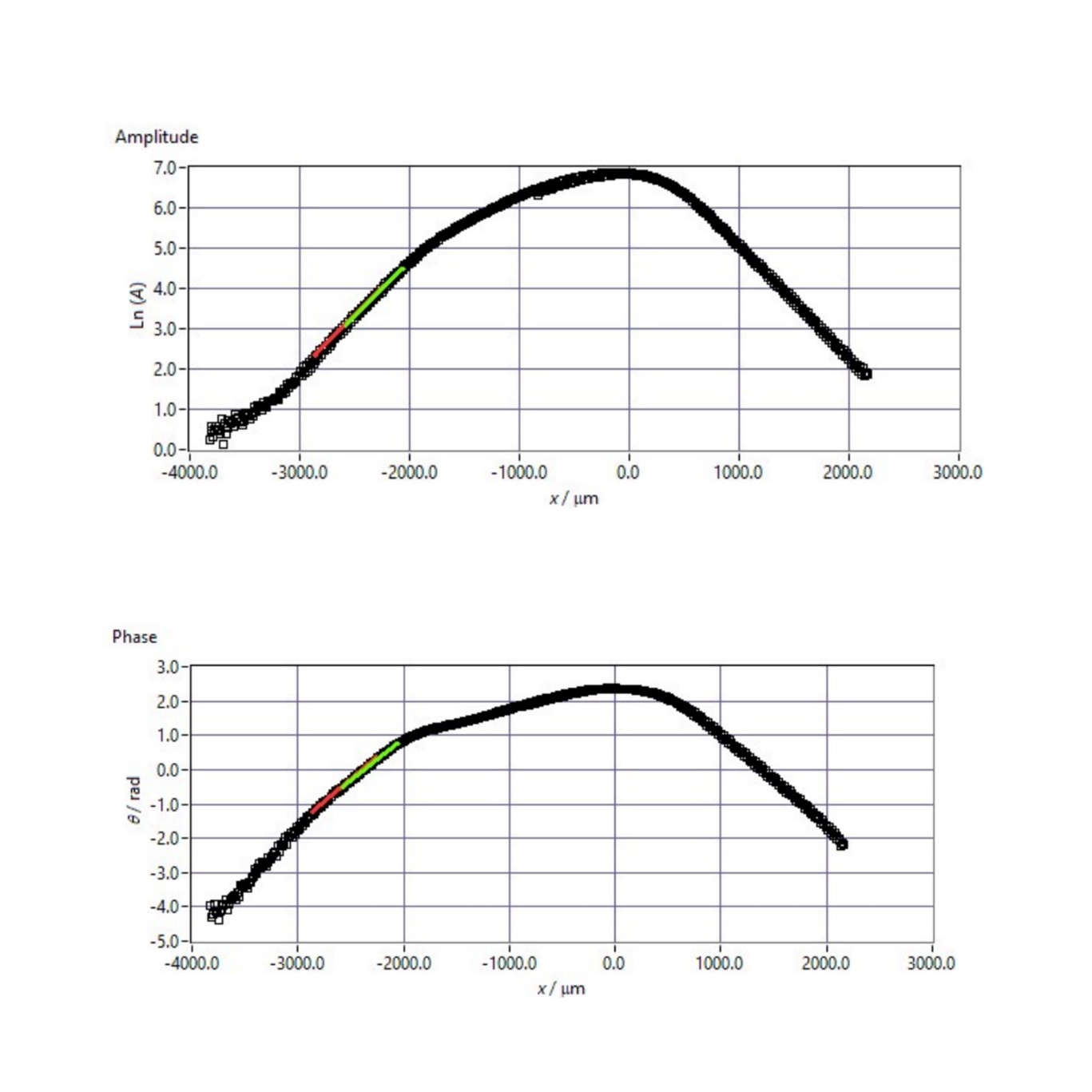
**

**Figure S30.** Diagram showing the distance dependence of the logarithmic amplitude and phase of the thermal diffusivity for the 10% Bi Mg_3_Bi_2_ thin film sample.


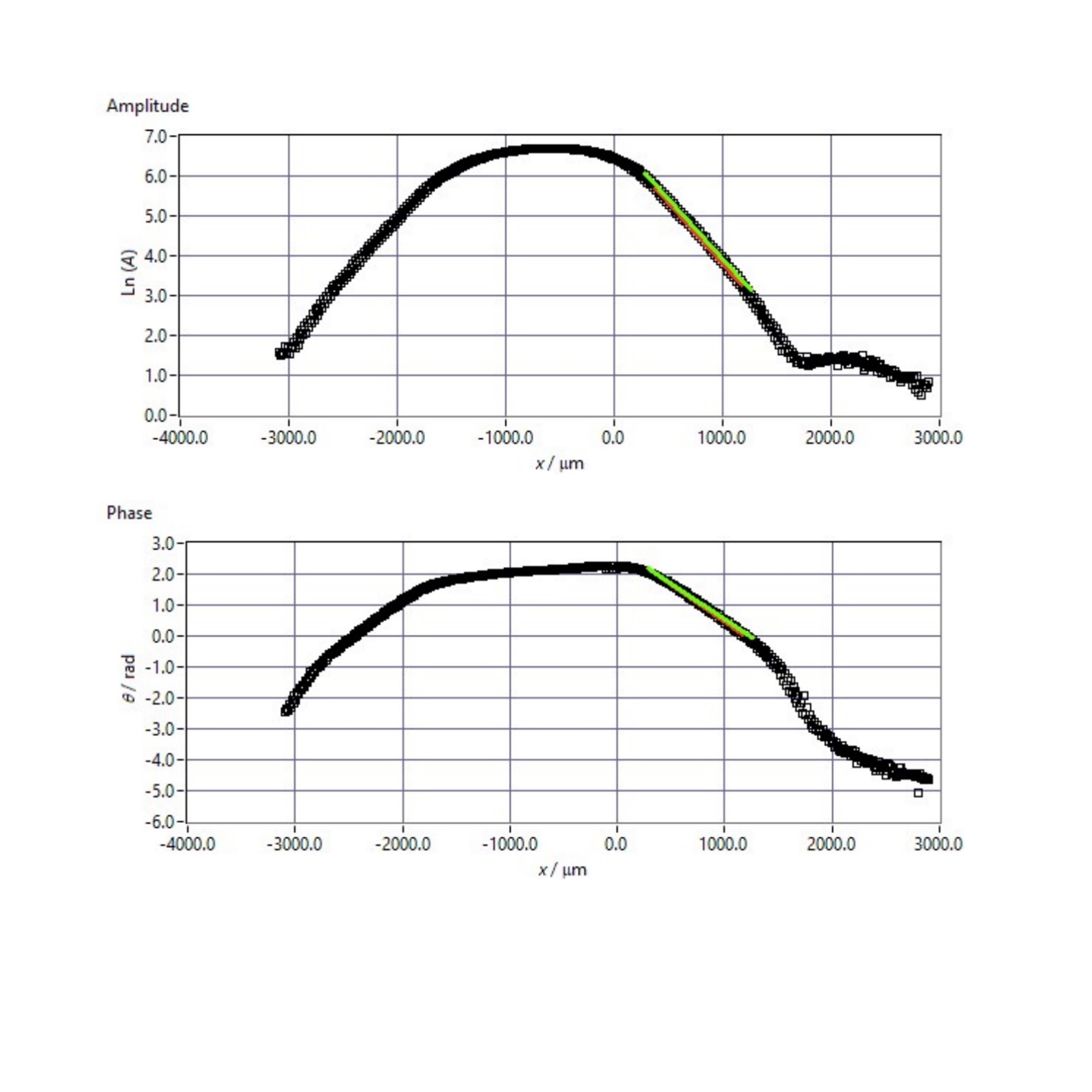


**Figure S31.** Diagram showing the distance dependence of the logarithmic amplitude and phase of the thermal diffusivity for the 28% Bi Mg_3_Bi_2_ thin film sample.

**
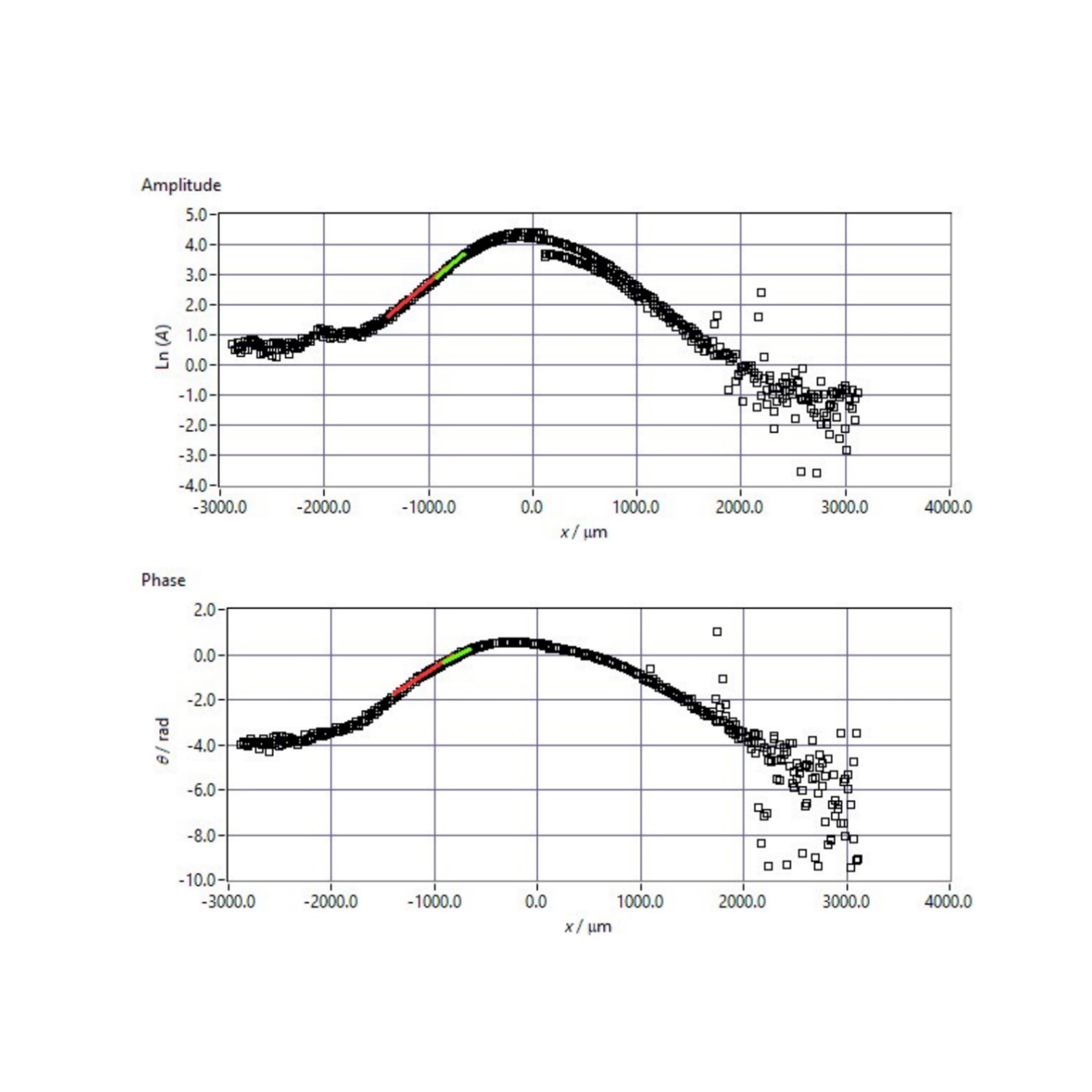
**

**Figure S32.** Diagram showing the distance dependence of the logarithmic amplitude and phase of the thermal diffusivity for the 40% Bi Mg_3_Bi_2_ thin film sample.

**
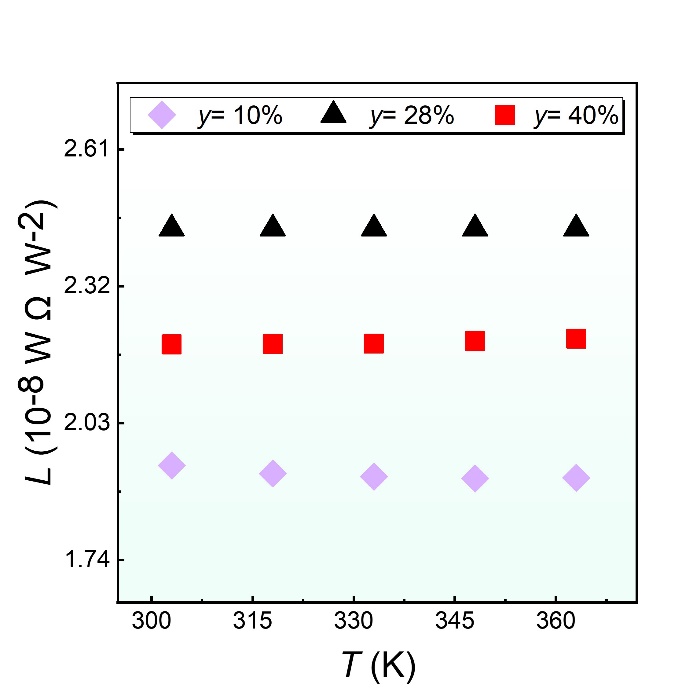
**

**Figure S33.** Lorenz parameter (*L*) calibration of Mg_3_Bi_2_ thin films with 10%, 28%, and 40% Bi.


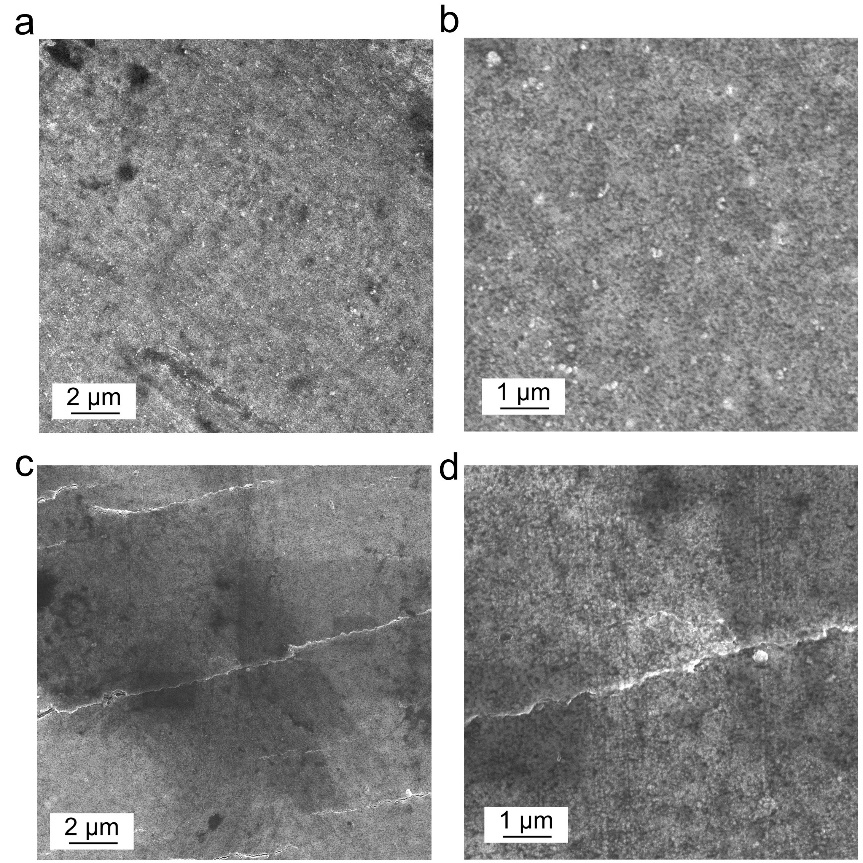


**Figure S34. SEM comparison before and after 500 bending of film with a radius of 5mm. a)** 3000 times image before bending. **b)** 10000 times image before bending. **c)** 3000 times image after bending. **d)** 10000 times image after bending.

**
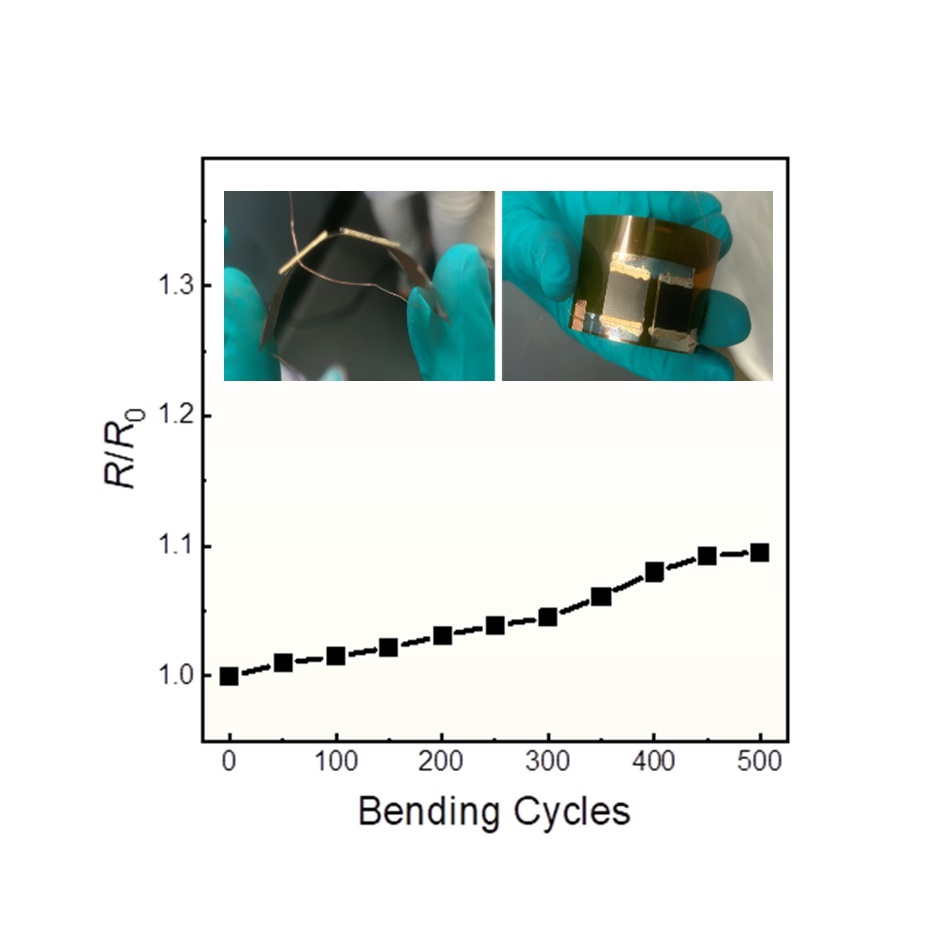
**

**Figure S35.** Resistance change (*R*/*R*_0_) of Mg_3_Bi_2_ device under different bending cycles with a bending radius of 10 cm.

##
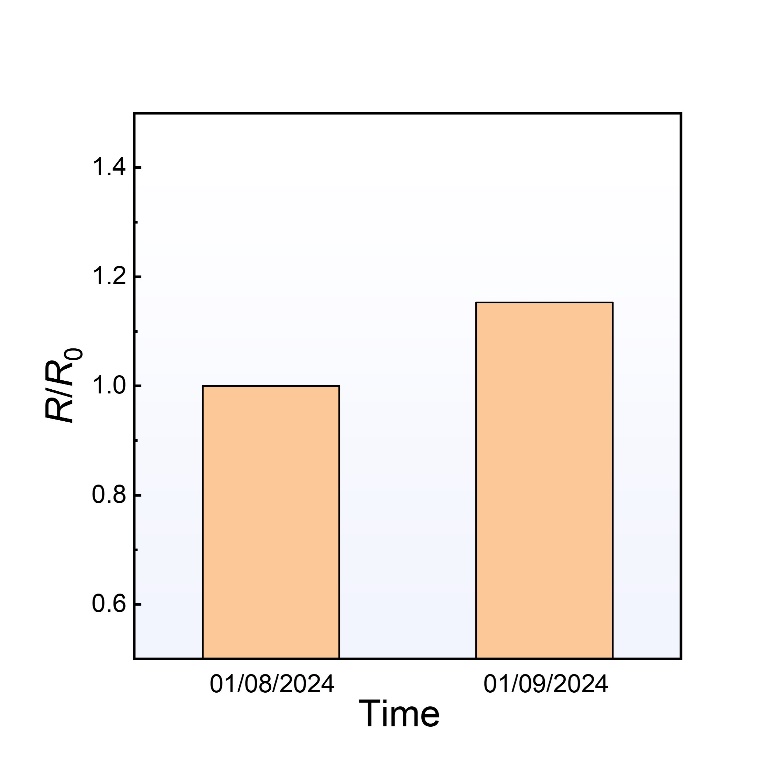


**Figure S36. *R*/*R*_0_ of Mg_3_Bi_2_ devices after one month of exposure to air.**

**Reference**

[1] Y. Xu, W. Li, C. Wang, J. Li, Z. Chen, S. Lin, Y. Chen, Y. Pei, *J. Mater. Chem. A* **2017**, *5*, 19143.

[2] J. Shen, Z. Chen, L. Zheng, W. Li, Y. Pei, *J. Mater. Chem. C* **2016**, *4*, 209.

[3] X. She, X. Su, H. Du, T. Liang, G. Zheng, Y. Yan, R. Akram, C. Uher, X. Tang, *J. Mater. Chem. C* **2015**, *3*, 12116.

[4] W. Liu, X. Shi, M. Hong, L. Yang, R. Moshwan, Z.-G. Chen, J. Zou, *J. Mater. Chem. C* **2018**, *6*, 13225.
